# A Modular Variable Stiffness Co‐Bot System Achieving Tasks Flexibility and Contact Compliance

**DOI:** 10.1002/advs.77372

**Published:** 2026-08-24

**Authors:** Wenlong Gaozhang, Yue Li, Jialei Shi, Yaxi Wang, Kaspar Althoefer, Agostino Stilli, Helge A. Wurdemann

**Affiliations:** ^1^ School of Computer Science and Electronic Engineering University of Essex Colchester UK; ^2^ Department of Computer Science University College London London UK; ^3^ Department of Mechanical Engineering Imperial College London London UK; ^4^ Department of Mechanical Engineering University College London London UK; ^5^ School of Engineering and Materials Science Queen Mary University of London London UK; ^6^ Department of Medical Physics and Biomedical Engineering University College London London UK

**Keywords:** human–robot interaction, hybrid robotic arm, modular robotics, variable stiffness actuation

## Abstract

Enhancing contact compliance and task flexibility is essential for expanding the real‐world use of robotic systems. This paper presents a modular collaborative robot system that combines antagonistic actuation with hybrid soft–rigid variable‐stiffness components, including a modular variable‐stiffness bending joint (mvsBJ), a modular variable‐stiffness rotational joint (mvsRJ), and a modular variable‐stiffness link (mvsL), supported by an integrated control framework. Experimental characterization shows that the mvsBJ achieves a 55.96° bending range and nearly threefold stiffness variation, from 9.16 to 24.72 Nm/rad, through fluidic pressure adjustment. The mvsRJ achieves 145° bidirectional rotation. The platform is benchmarked across repeated trials in healthcare assistance and industrial support scenarios. In an assistive feeding task, a 3‐DoF RBBL configuration operates in a low‐stiffness mode, maintaining bounded force‐tracking variation with mean error drift below 0.16 N. Collision tests across 40–60 mm/s show peak interaction forces increasing predictably from 3.93 to 5.38 N. In an industrial chamfering task, the 2‐DoF RB configuration achieves pressurized rigidity comparable to a conventional mechanical workbench. These results demonstrate plug‐and‐play reconfigurability with reliable adjustable compliance, providing a novel architecture for diverse physical human–robot collaboration applications.

## Graphical abstract

Bio‐inspired antagonistic compliance enables a modular rigid‐soft co‐bot platform with variable stiffness for safe human‐robot interaction (HRI): Standardized interfaces support application‐driven configurations with tailored DoFs, workspace, and stiffness range, making the system suited to HRI and low‐volume automation. Fluidic pressure control enables touch‐safe assistive feeding and workbench rigidity for machining, demonstrating a scalable robotic paradigm for healthcare and industrial manufacturing, for instance.

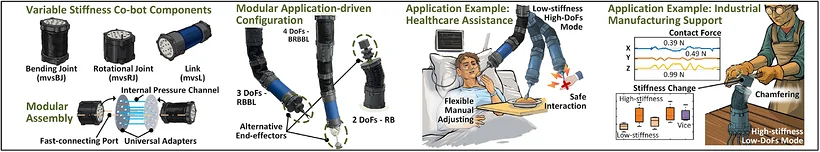

## 1 Introduction

In recent years, lightweight robots designed to collaborate with humans—commonly known as collaborative robots (co‐bots)—have seen significant adoption across diverse domains [1, 2]. For example, in industrial production, co‐bots have played a pivotal role in facilitating the implementation of Industry 4.0, assisting workers in tasks such as material handling and assembly [3]. Beyond industry, they are increasingly used in everyday services to automate routine activities, improving both efficiency and convenience [4]. During the COVID‐19 pandemic, for instance, a substantial number of co‐bots were deployed as autonomous sampling devices to ensure the safety of healthcare personnel by reducing exposure risks [5, 6]. In research environments, co‐bots are often integrated into experimental setups, contributing to the automation of experiments [7]. A major factor driving the widespread adoption of cobots in these areas is their exceptional focus on ensuring human safety, especially when compared to traditional robots [8]. This focus on safety enables close human‐co‐bot interaction in the workspace, promoting effective and efficient collaboration [9].

Contemporary co‐bots are predominantly motor‐driven, with safety ensured through a range of sensors – such as force/torque sensors and distance sensors—and complex control algorithms [10]. However, the complexity of these safety measures introduces the potential for failure due to uncontrollable factors, which could compromise human safety [11]. Additionally, while high degrees of freedom allow for more flexible motion paths, they also increase the cost and control complexity of co‐bots [12]. In some applications, only low degrees of freedom are necessary to complete tasks effectively. Therefore, enabling easy configuration of a co‐bot's structure – such as adjusting its degrees of freedom or size – according to task‐specific requirements can significantly enhance their efficiency while reducing both cost and control complexity.

In the current landscape of co‐bot research, a wide range of software‐ and hardware‐based safety measures have been proposed. On the software side, methods such as impedance control [13, 14, 15] and software‐based automatic safety controllers (ASCs) [16] have been introduced to reduce stiffness or halt robot motion in emergencies. However, these methods depend heavily on algorithms and sensors, which are prone to occasional failures or malfunctions – posing safety risks that must be carefully managed. From a hardware perspective, developing components that can directly alter stiffness has proven to be a more robust solution. Various stiffness‐variable joints [17, 18, 19, 20, 21] and stiffness‐variable links [22, 23, 24, 25, 26, 27, 28] have been developed, allowing stiffness to be adjusted by limiting the stiffness of joints or links, thereby reducing the potential for injury during system failures. Yet, current stiffness‐variable components often rely on metal elastic components, such as springs and leaf springs, which results in excessive overall mass and volume. This is not conducive to achieving safer human‐robot interaction. To address these limitations, researchers have begun incorporating soft components for stiffness modulation [29, 30, 31, 32, 33, 34, 35]. Soft actuators, for instance, have been proposed and integrated into robot arms [36]. However, soft materials introduce challenges such as nonlinear behavior, hysteresis, and buckling [37, 38, 39, 40, 41], along with limitations in load‐bearing capacity [42]. Therefore, finding the right balance between the advantages and disadvantages of soft materials is a significant challenge. In the realm of modular design, extensive research has demonstrated that modularity and reconfigurability can provide robots with unique flexibility and improved efficiency [43, 44, 45, 46, 47]. Applying this design philosophy into co‐bot design offers the potential for customizable, task‐specific configurations, further improving their flexibility and efficiency in diverse environments. Stiffness modulation in compliant and soft robotics can be achieved through various state‐of‐the‐art approaches, notably material‐phase‐transition technologies, granular/layer jamming mechanisms, and antagonistic actuation structures. Jamming mechanisms, for example, offer effective ways to dynamically alter the rigidity of grippers or joints by compacting localized sheets or particles under vacuum or positive pressure. Alternatively, drawing inspiration from biological systems like the human musculoskeletal apparatus or the muscular hydrostats of an octopus arm, antagonistic fluidic actuation utilizes opposing active chambers to modulate joint impedance without relying on hard state transitions. This principle has been successfully incorporated into advanced hybrid soft‐rigid platforms, such as those presented by Chen et al. [48], who developed reconfigurable components combining soft muscle elements with mechanical frameworks, and the extensive research by Manti et al. [49] and the Sant'Anna group [50, 51, 52] on integrating compliant elements into rigid joints for safer interaction. A notable commercial implementation of fluidic antagonistic actuation is the Festo BionicCobot [53]. While the BionicCobot utilizes double‐acting pneumatic vane actuators to mimic human arm motion, it relies on a fixed, non‐reconfigurable structure with permanent exterior lines. Our system advances this paradigm by offering fully modular, plug‐and‐play components. By embedding a multi‐channel pressure bus directly within universal mechanical interfaces, our design allows users to customize degrees of freedom (DoF) on‐demand while avoiding external line clutter, mirroring early flexible pneumatic designs like the multi‐DoF actuator proposed by Bao et al. [54], but with the added layer of structural modularity.

**FIGURE 1:**
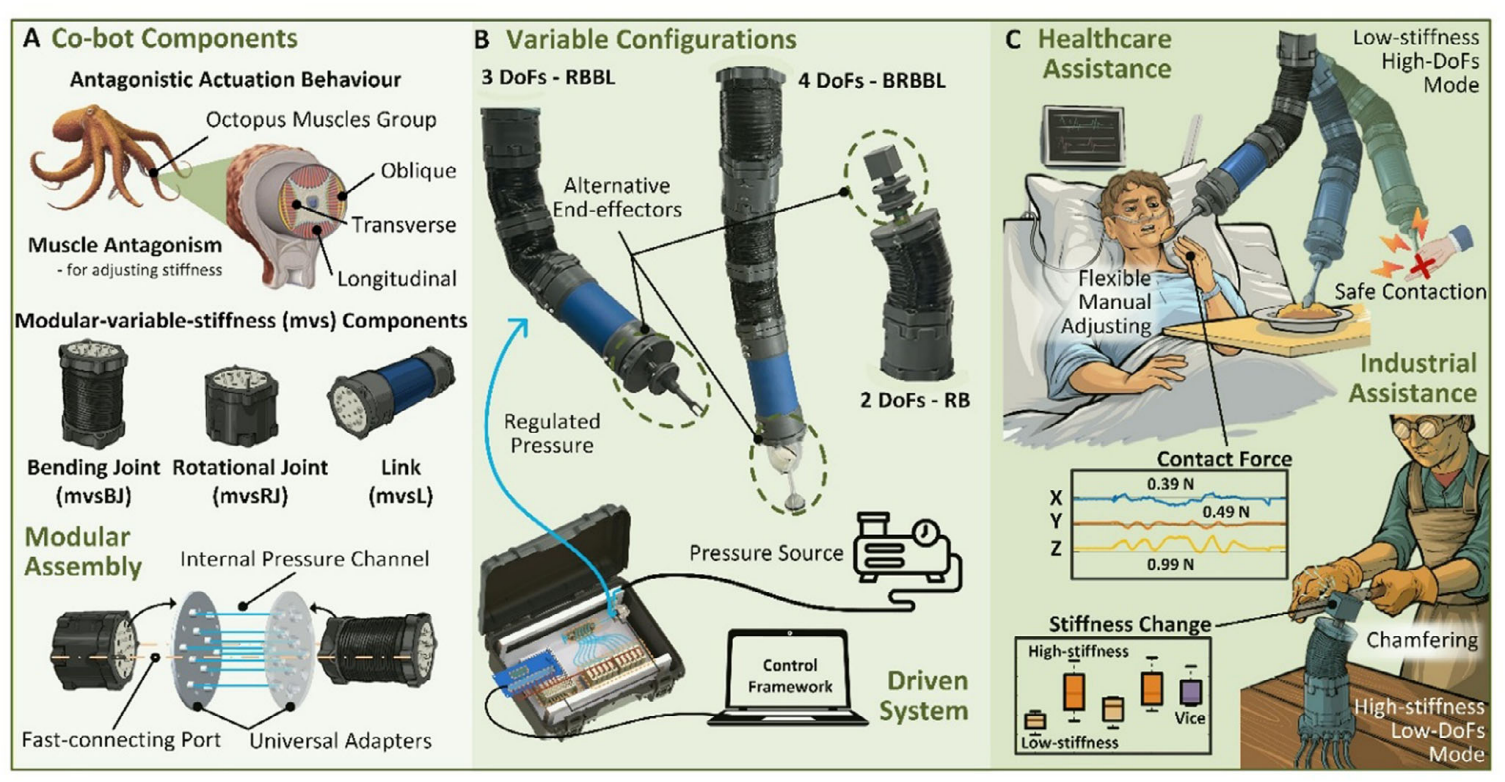
Introduction of the modular variable stiffness co‐bot system. (A) Bio‐inspiration from octopus muscle groups exhibiting antagonistic actuation for variable stiffness control. This principle is adopted in the design of modular variable stiffness (mvs) co‐bot components, including the bending joint (mvsBJ), rotational joint (mvsRJ), and link (mvsL). The components can be rapidly assembled into various co‐bot configurations through universal adapters, integrated pressure channels, and quick‐connect ports. (B) By selecting different types and numbers of components, modular variable stiffness co‐bots with 2‐, 3‐, and 4‐DoF configurations can be assembled and equipped with alternative end‐effectors to meet diverse task requirements. A unified actuation system, comprising the control framework and pressure regulation unit, enables consistent control across all co‐bot configurations. (C) Two application scenarios—healthcare and industrial assistance—were conducted to demonstrate the flexibility and safety of the proposed co‐bot system. In the healthcare assistance scenario, a 3‐DoF co‐bot was employed to deliver food from the table to a position near the patient's mouth. The patient could then manually adjust the end‐effector to bring the food to their mouth with minimal effort, as the co‐bot operated in a low‐stiffness mode. The recorded contact forces indicated that the average force along all three axes was less than 1 N, confirming the system's safety during human interaction. In the industrial assistance scenario, a 2‐DoF co‐bot was used to hold a workpiece, providing a flexible yet stable working position under a high‐stiffness mode. The measured holding stiffness demonstrated that the proposed antagonistic stiffness adjustment method significantly enhanced the overall rigidity of the co‐bot, reaching a stiffness level comparable to that of a conventional workbench.

To better position this work within the state of the art, the proposed framework is explicitly benchmarked against these existing modular, soft, and variable‐stiffness robotic technologies. Traditional variable‐stiffness robots typically rely on rigid metallic components, such as leaf springs or rotary flexures, which inherently increase system mass and volume, limiting safe human‐robot interaction. On the other hand, while fully soft robotic architectures offer compliance, they frequently suffer from low payload capacity and structural buckling under load. Our hybrid rigid‐soft approach bridges this gap by leveraging standardized rigid skeletons for structural alignment alongside antagonistic soft fluidic chambers for active stiffness modulation. Crucially, unlike conventional modular soft robots that necessitate complex external tubing networks for each added module, the co‐bot developed in this study embeds a continuous, standardized 10‐channel internal pneumatic pathway through universal mechanical adapters. This architecture allows for rapid, seamless plug‐and‐play topological reconfiguration while consistently sustaining uniform pressure distribution across high‐DoF assemblies, enabling the system to comprise stiffness‐variable bending joints, stiffness‐variable rotational joints, stiffness‐variable links, and flexible end‐effectors without the need for external tubing between modules (see Figure 1A). Inspired by the shared biological mechanism of muscle antagonism, all components can actively modulate stiffness through fluidic regulation. This structural modularity allows users to flexibly select component types and quantities according to specific task requirements, thereby facilitating the construction of customized, task‐specific co‐bot configurations that significantly enhance operational efficiency while reducing global system costs, as illustrated in Figure 1B.

To validate the advantages of the proposed reconfigurable co‐bot system, two representative application scenarios were implemented, highlighting its unique flexibility and wide range of variable stiffness, as depicted in Figure 1C. The first scenario focuses on healthcare assistance, where a three‐degree‐of‐freedom (3‐DoF) co‐bot delivers food from a table to a position near a patient's mouth. This configuration allows a patient with limited mobility to manually guide the end‐effector and feed themselves. Operating in an intrinsically low‐stiffness mode, the co‐bot ensures safe and intuitive physical interaction, making it particularly suitable for delicate healthcare tasks. The second scenario demonstrates industrial assistance, where a two‐degree‐of‐freedom (2‐DoF) co‐bot securely holds a workpiece, effectively functioning as a reconfigurable and compliant workbench. When operating in a high‐stiffness mode, the co‐bot provides sufficient rigidity to support tasks such as chamfering or assembly, achieving performance levels comparable to those performed on a traditional fixed workbench. Furthermore, its ability to autonomously reorient the workpiece enhances operational flexibility and reduces manual workload. Together, these two scenarios underscore the co‐bot's adaptability across diverse application domains, seamlessly integrating safety, flexibility, and functionality in both healthcare and industrial environments.

## 2 Results

### 2.1 Modular Variable Stiffness Components

#### 2.1.1 Modular Stiffness Variable Bending Joints (mvsBJs)

**FIGURE 2:**
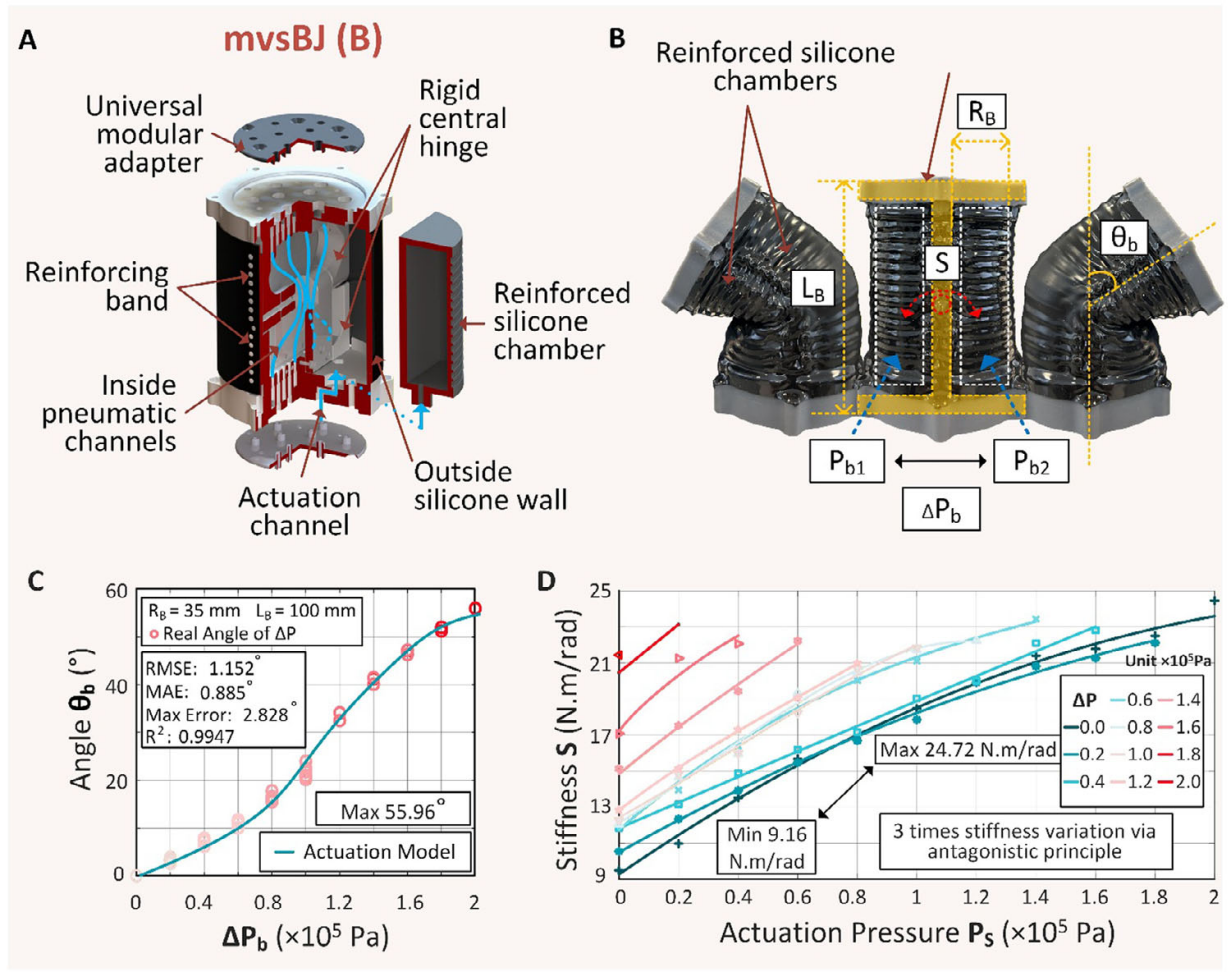
Structural design and quantitative characterization of the modular variable‐stiffness bending joint (mvsBJ). (A) Sectional CAD layout demonstrating the internal configuration, including pneumatic actuation channels, central flexible hinge, and fiber‐reinforced silicone chambers. (B) Physical prototype demonstrating the multi‐directional bending motion profile governed by the differential actuation pressure ΔP_b_. (C) Quantitative validation curve mapping the bending angle θb against the actuation pressure differential ΔP_b_, directly contrasting the experimental data points with the smooth trajectory calculated from the analytical actuation model alongside explicit error metrics (RMSE = 1.152°, MAE = 0.885°, Max Error = 2.828°, R^2^ = 0.9947). (D) Joint stiffness S characterization curves plotted as a function of the primary actuation pressure Ps across various tracking boundaries (ΔP), demonstrating a wide‐range 3‐fold stiffness variation via the antagonistic fluidic regulation principle.

Drawing inspiration from the human musculoskeletal system, we present a novel design for modular stiffness‐variable bending joints (mvsBJs) based on the antagonistic actuation principle. The mvsBJ (as shown in Figure 2A) features a rigid central hinge, analogous to the human skeleton, flanked by two semi‐circular, fiber‐reinforced silicone rubber chambers that mimic muscle function. The rigid hinge serves as the rotation center and constrains the axial direction of the soft chambers, ensuring controlled bending. To concentrate the actuation forces to drive bending motions at the hinge, a discrete reinforcing band, fabricated by winding a high‐strength fiber thread continuously in a helical fashion around the exterior wall, is utilized to limit radial expansion while maintaining high axial bending flexibility.

The joint achieves bending by selectively inflating one chamber, which produces a commanded bending angle. When both chambers are inflated simultaneously, the opposing forces generated by the chambers interact antagonistically, enabling precise control of joint stiffness. This mechanism closely resembles the human musculoskeletal system, which modulates stiffness to adapt to varying operational demands: achieving higher stiffness for tasks requiring accuracy or heavy payload handling and reducing stiffness for safe and compliant interactions. To support modular integration, the joint design incorporates fully embedded pneumatic channels and universal modular adapters at both ends, facilitating rapid connection with other robotic components.

Based on the above design concept, we fabricated, modeled, and characterized an mvsBJ prototype (as shown in Figure 2B). By applying the dimensions of the mvsBJ prototype to the analytical model, we derived the bending angle curve under the different actuation pressure ΔP_b_ (as shown in Figure 2C). The physical prototype characterization shows the mvsBJ prototype achieved 55.96 degrees of movement in each direction by controlling the actuation pressure ΔP_b_, which aligns with the results from the modeling. Furthermore, the stiffness variation characterization results (as shown in Figure 2D) exhibited about threefold stiffness variation by controlling the pressure PS (i.e., from 9.16 to 24.72 Nm/rad), confirming the effectiveness of the antagonistic actuation principle for stiffness control.

#### 2.1.2 Modular Stiffness Variable Links (mvsLs)

**FIGURE 3:**
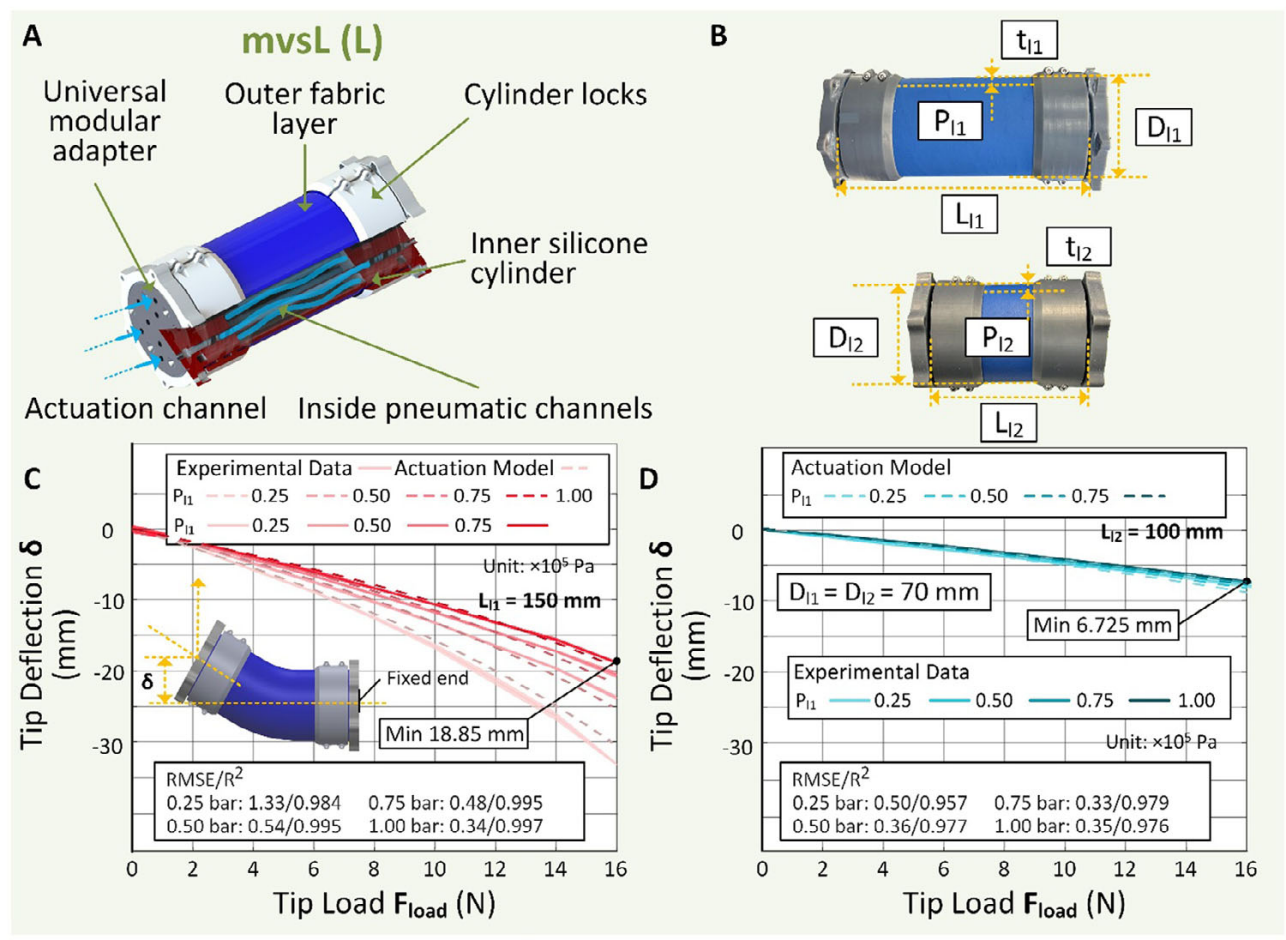
Mechanical architecture and predictive tip deflection models for the modular variable‐stiffness link (mvsL). (A) Detailed cross‐sectional CAD rendering displaying the outer fabric layer, internal fluid channels, and nested silicone cylinder configurations. (B) Physical prototypes fabricated with distinct parameter constraints, delineating variations in active length (L_l_) and body diameter (D_l_). (C) Transverse tip deflection δ plotted against a dynamic tip load spectrum (Fload from 0 to 16 N) for the longer link prototype (L_l1_ = 150 mm), providing a continuous overlay comparison between raw experimental metrics and the analytical actuation model predictions across multiple pressure thresholds (P_l1_ from 0.25 to 1.00 bar) along with the corresponding error matrix. (D) Quantitative tip deflection validation profiles for the shorter link variant (L_l2_ = 100 mm) under identical loading boundaries, confirming tight model fitting accuracies with specific RMSE and R^2^ correlation metrics annotated for each active pressure state.

In line with the mvsBJs, modular variable stiffness links (mvsLs) are designed to achieve stiffness control based on the antagonistic actuation principle. The mvsL features a cylindrical inner layer constructed from silicone rubber and an outer reinforcement layer made from non‐stretchable fabric, as shown in Figure 3A. Unlike the helical band thread used in the bending joints, this reinforcement layer consists of a continuous sheet of non‐stretchable woven fabric wrapped completely around the cylinder. When the inner pressure of the silicone cylinder is increased, the resulting expansion is securely constrained by this outer reinforcement layer, creating a counteracting force that significantly enhances the global stiffness of the link without shifting its central axis. This stiffness variability allows the mvsL to adapt to different task requirements. In high‐stiffness mode, it supports high‐precision manipulation or heavy‐load tasks; in low stiffness‐mode, it facilitates safe, compliant interaction or flexible adaptability. To enable universal modularization, the design integrates embodied pneumatic channels and universal modular adapters at both ends as well, facilitating rapid and seamless connection with other robotic components.

To validate this concept, we fabricated two mvsL prototypes with lengths of 150 and 100 mm (shown in Figure 3B), respectively, and characterized their tip deflection under varying tip loads (0 to 16 N) and internal tuning pressures (0 to 1 × 10^5^ Pa). The quantitative evaluation metrics are distinctively illustrated in Figure 3C (for the 150 mm link) and Figure 3D (for the 100 mm link). The results demonstrate that increasing the inner pressure significantly reduces tip deflection across all loading profiles, formally confirming the high‐efficiency stiffness modulation capability of the design. For instance, under the maximum extreme load of 16 N, the 150 mm mvsL prototype operating at baseline unpressurized conditions exhibited a pronounced deflection; however, when the internal pressure was raised to a maximum of 1.00 × 10^5^ Pa, the maximum tip deflection was securely suppressed to a minimum boundary of 18.85 mm. In comparison, under the identical 16 N load condition, the shorter 100 mm mvsL prototype demonstrated a drastically enhanced structural rigidity, effectively limiting its maximum end deflection to a mere 6.725vmm at full pressurization. This scaling phenomenon confirms that shorter mvsL structures inherently exhibit greater baseline stiffness under equivalent pneumatic and load inputs. Overall, these empirical findings quantitatively prove that the proposed mvsLs can successfully achieve a rapid, reversible switch between high‐stiffness and low‐stiffness modes, aligning tightly with the targeted design objectives for tasks flexibility and contact compliance.

#### 2.1.3 Modular Stiffness Variable Rotation Joints (mvsRJs)

**FIGURE 4:**
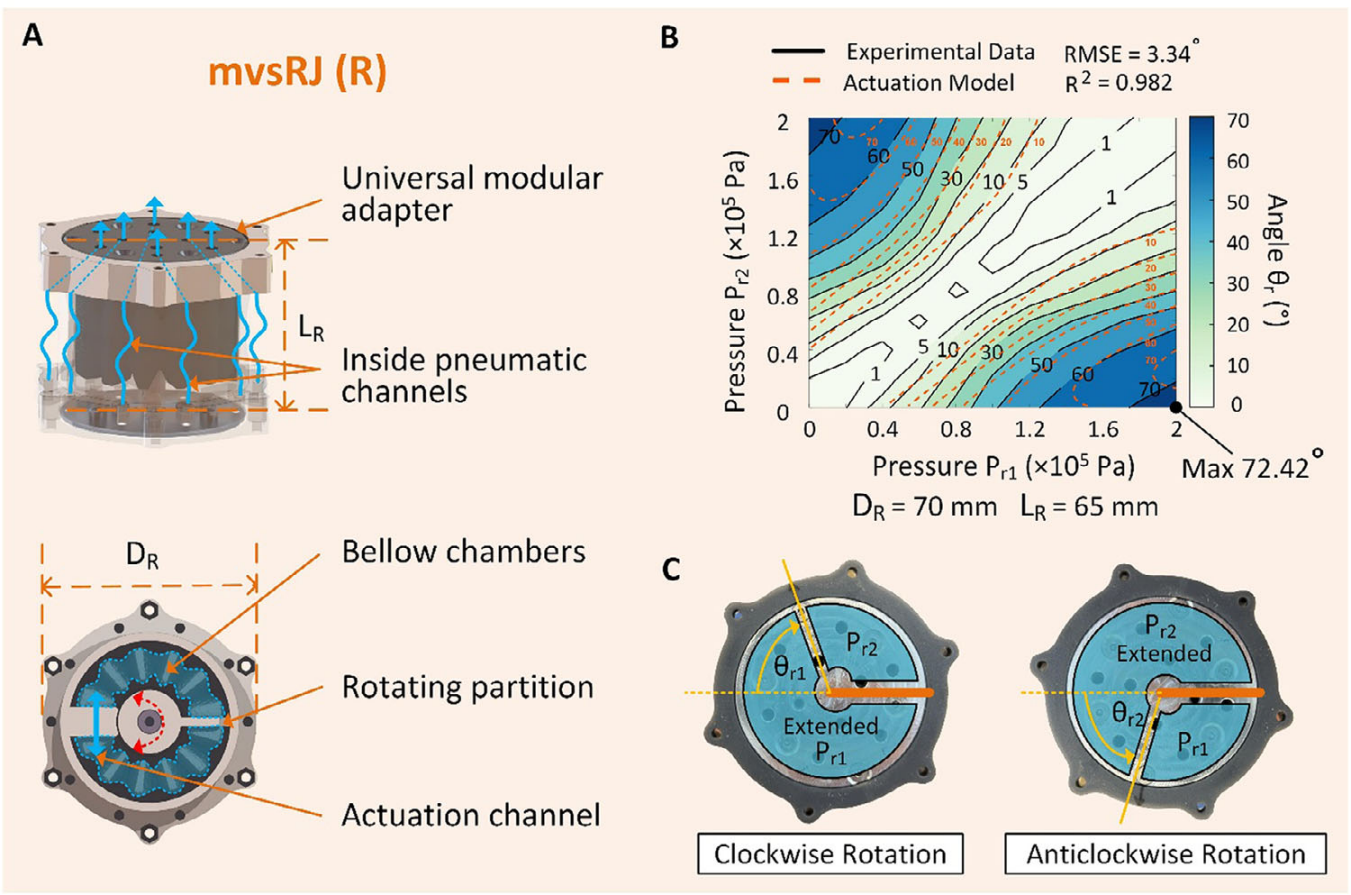
Layout configuration and angular mapping models of the modular variable‐stiffness rotational joint (mvsRJ). (A) Exploded CAD view and internal radial cross‐section highlighting the layout of the multi‐segment internal bellow chambers, rotating partition wall, and modular universal adapters. (B) Integrated bi‐directional rotation angle (θr) response mapping contour under varied multi‐channel pneumatic inputs (P_r1_ and P_r2_), verifying a high predictive convergence between the empirical measurements (solid contour lines) and the proposed analytical actuation model (dashed orange contours) with a bounded global RMSE of 3.34° and an R^2^ of 0.982. (C) Physical actuation prototypes displaying explicit clockwise and anticlockwise angular deflections under differential pressure expansion.

While the mvsBJs can achieve two‐dimensional (X and Y axes) motion capability by altering their assembly direction, enabling three‐dimensional motion capability for the co‐bot system requires a joint capable of rotation around the Z‐axis. To address this, we developed modular variable‐stiffness rotational joints (mvsRJs), extending the same antagonistic actuation and modular design principles used in the mvsBJs, but replacing the hinge with a rotational shell and plate mechanism as shown in Figure 4A.

The mvsRJ is actuated by two bellow‐shaped, fiber‐reinforced silicone rubber chambers. The expansion of each chamber independently drives the rotational plate to achieve rotation in opposite directions, while simultaneous inflation of both chambers increases the stiffness of the joint as shown in Figure 4B. As with other components, the design incorporates fully integrated pneumatic channels and universal modular adapters to facilitate modular assembly with other system elements.

We fabricated an mvsRJ prototype and characterized its rotational performance. As detailed in the experimental actuation map in Figure 4C, the relationship between the dual‐chamber fluidic pressures P_r1_ and P_r2_) and the resulting rotational deflection (θ_*r*_) is mapped comprehensively. The results indicate that the mvsRJ can achieve a maximum clockwise or anticlockwise rotation angle of 72.42°per chamber, yielding a total bidirectional rotation range of 145° under an actuation pressure of 2 bar. The contour distributions further reveal an asymmetric angular boundary that is highly dependent on the pressure combinations of the two antagonistic chambers, validating its capacity to function reliably within the co‐bot system.

#### 2.1.4 Prototype Family and Co‐Bots With Different Configurations

**FIGURE 5:**
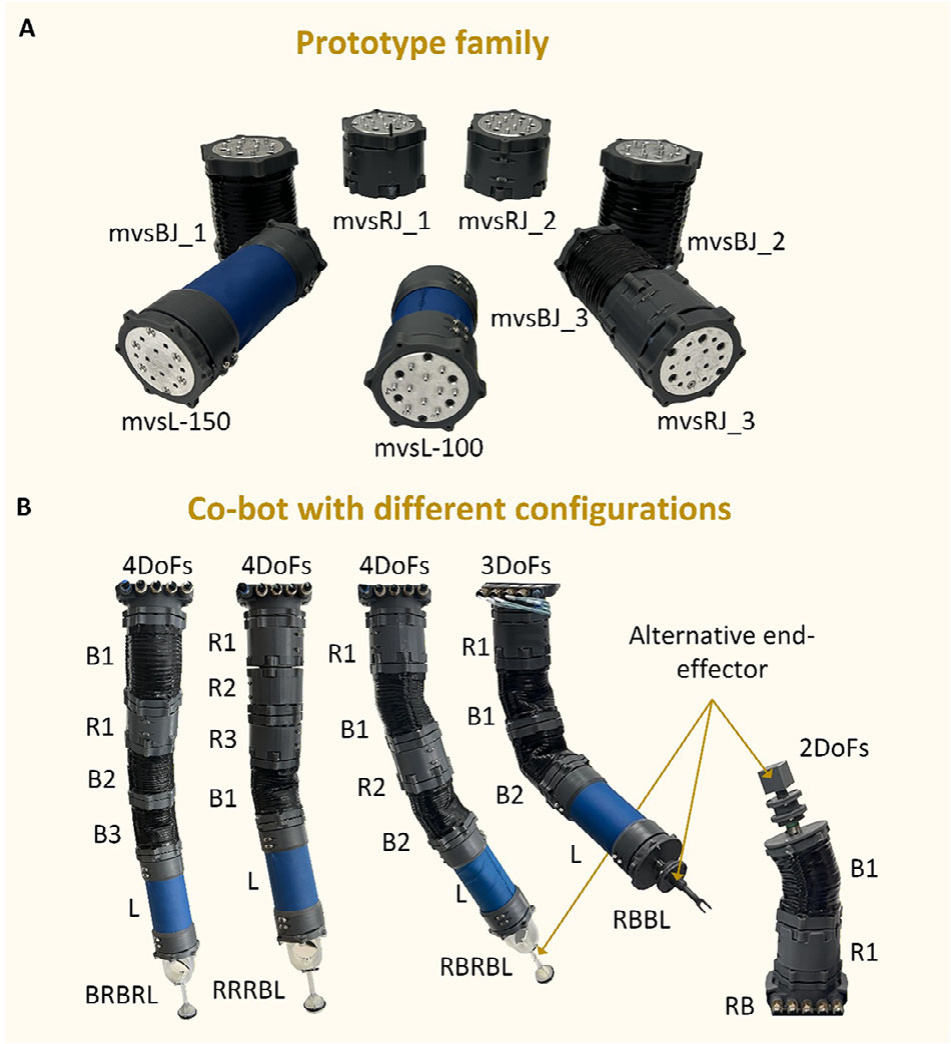
Comprehensive component ecosystem and multi‐DoF architectural configurations of the customizable co‐bot platform. (A) The complete hardware prototype family, showcasing the fabricated modules with varied specifications and geometric boundaries (including mvsBJ, mvsRJ, and mvsL series). (B) Demonstrations of five representative reconfigurable co‐bot topologies assembled via universal modular adapters, spanning distinct degrees of freedom (from a highly agile 4‐DoF arm down to a compact 2‐DoF system) equipped with alternative customized end‐effectors for safety‐oriented automation tasks.

To construct the complete co‐bot system, we fabricated a prototype family (Figure 5A) comprising two mvsBJs, two mvsRJs, and two mvsLs. These prototypes include a total of 10 integrated pneumatic channels, enabling the assembly of up to four joints and two links to form co‐bots with various configurations. As demonstrated in Figure 5B, based on the fabricated prototype family, we successfully built five co‐bot prototypes of different topologies—namely BRBRL, RRRBL, RBRBL, RBBL, and RB—which span across 2, 3, and 4 degrees of freedom (DoFs). These physical implementations highlight the system's structural modularity and adaptability, allowing co‐bots to be tailored to diverse tasks and operational requirements.

#### 2.1.5 Modular Design for Modules and Internal Pressure Channels Connection

**FIGURE 6:**
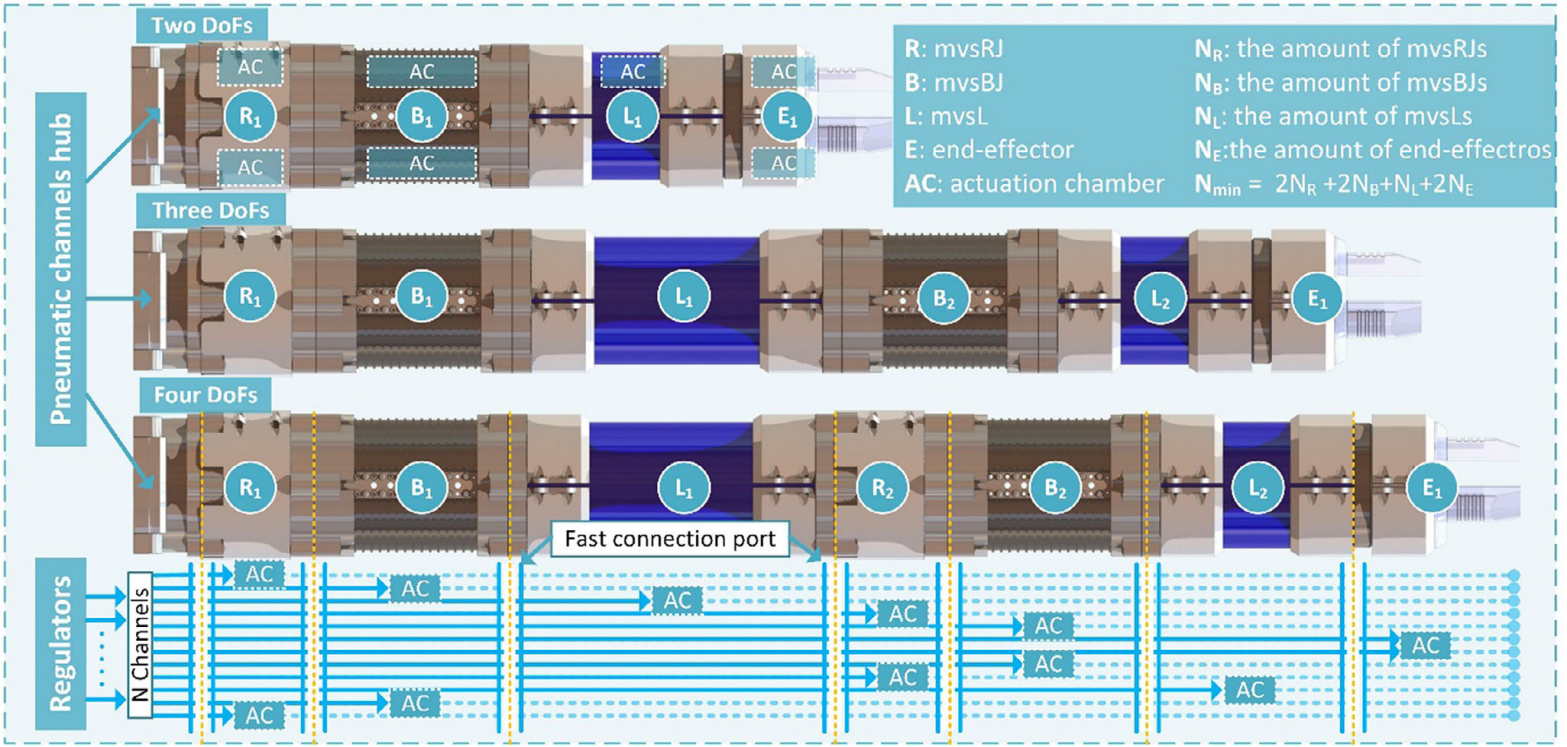
Modular pneumatic routing and mechanical interface design. Each module (e.g., mvsBJ, mvsRJ, and mvsL) is equipped with a universal modular adapter featuring male and female ends of standardized diameter, enabling direct axial connection. Bolt–nut ports distributed around the interfaces ensure alignment and apply clamping force, compressing internal O‐rings to seal the joint. All modules include 10 internal pneumatic channels (visualized as blue lines), allowing continuous airflow throughout the assembled co‐bot regardless of configuration. A pneumatic hub at the base provides external tube connections via quick push‐in fittings and passes the 10 channels upward through a male adapter for seamless integration. This architecture ensures both modularity and uninterrupted pressure routing across complex co‐bot assemblies.

To facilitate continuous reconfigurability without external fluidic entanglement, a standardized and self‐contained mechanical‐pneumatic coupling framework is implemented across all modules as shown in Figure 6. All modular bending joints (mvsBJs), rotational joints (mvsRJs), and links (mvsLs) are equipped with universal mechanical adapters featuring precision male and female concentric interfaces of identical diameter to enable straightforward axial coupling. To secure this mechanical connection, each component integrates evenly distributed bolt–nut ports around both interfacing faces. These ports serve a dual functional purpose: (i) ensuring precise structural alignment and angular orientation during topological assembly, and (ii) applying a controlled, uniform compressive clamping force that deforms localized rubber O‐rings embedded within the interface tracks, establishing an airtight pneumatic seal capable of safely withstanding operating pressures up to 2 bar.

To support flexible reconfiguration and guarantee uninterrupted pneumatic continuity, each module is equipped with the full set of *N* internal pressure channels, where *N* is the maximum number of channels needed by any co‐bot configuration. In this work, we standardize *N*  =  10, as visualized by the blue pneumatic lines in Figure 6. These channels are routed through every module regardless of whether a particular module utilizes all of them. At the system base, a pneumatic hub houses quick‐connect air tube fittings that interface with all *N* channels. The hub itself features a male modular adapter, allowing direct connection to other components in the stack. As a result, the full assembly maintains up to 10 uninterrupted pneumatic pathways across all components. Each actuatable chamber in the co‐bot can be assigned a dedicated channel, avoiding supply conflicts between modules.

To determine the minimum number of pneumatic channels *N_min_* required for a specific co‐bot configuration, we define:

$$N_{min}=2N_{B}+2N_{R}+N_{L}+N_{E}$$

where *N_B_*, *N_R_*, *N_L_*, and *N_E_* denote the numbers of mvsBJ mvsRJ mvsL, and end effectors, respectively. This modular routing and standardization strategy enables scalable and reconfigurable co‐bot design.

### 2.2 Healthcare Scenario: Co‐Bot RBBL for Feeding Assistance

**FIGURE 7:**
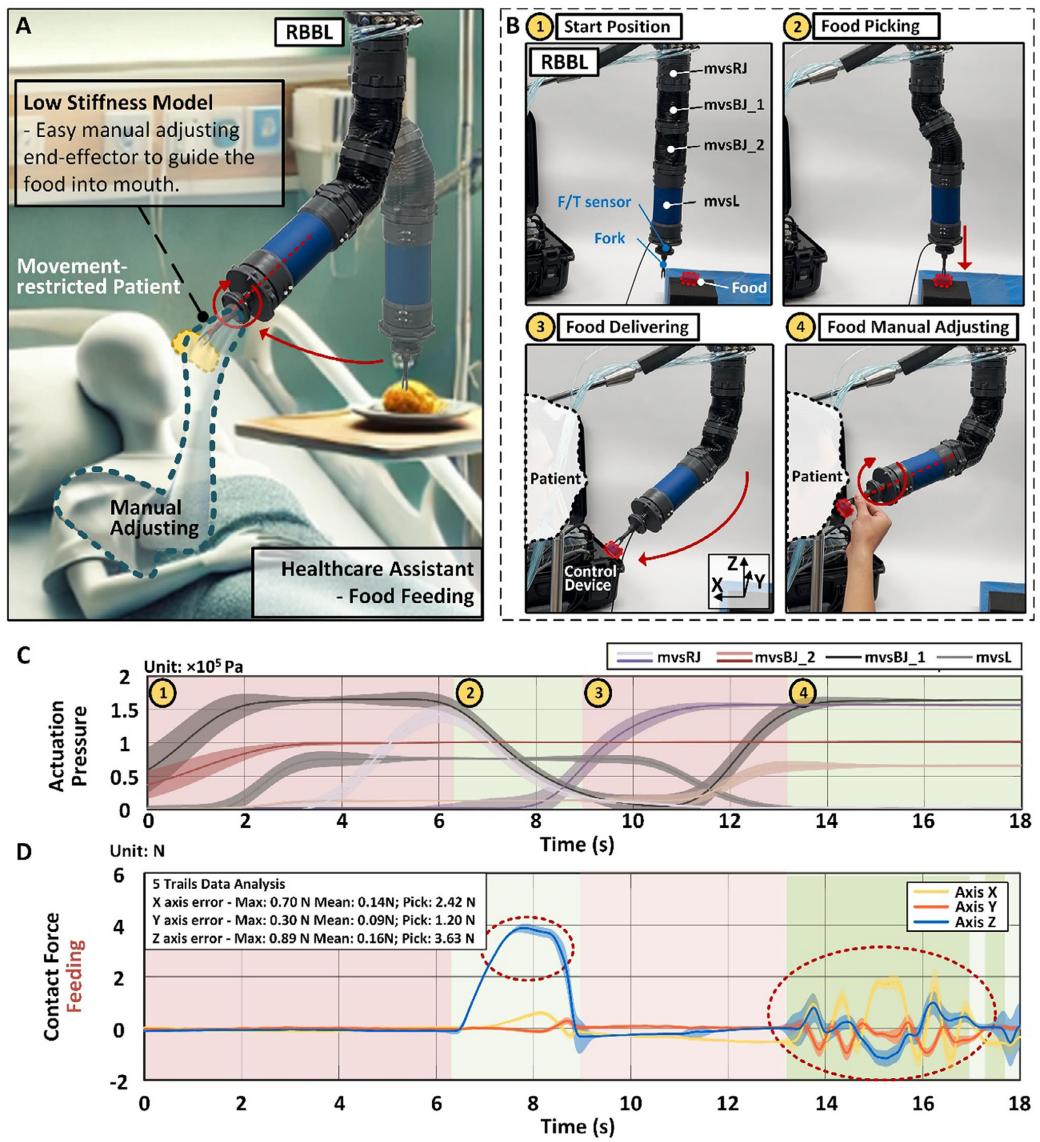
Healthcare application demonstration and statistical characterization of the co‐bot during a food‐feeding task. (A) Conceptual operational schema of the 4‐DoF RBBL configuration assisting a movement‐restricted patient via low‐stiffness manual adjustment modes. (B) Chronological physical snapshots delineating the four key operational phases: (1) start position, (2) food picking, (3) food delivering, and (4) manual trajectory adjustment. (C) Statistical multi‐channel actuation pressure profiles under five independent repeated trials (*n* = 5), plotted as the Mean trajectory enclosed by a continuous Standard Deviation (± SD) shaded error band. (D) Integrated multi‐axis contact force response profiles (Mean ± SD shaded bands) across the X, Y, and Z trajectories, featuring annotated quantitative peak forces and tracking error matrices.

The first application scenario focuses on healthcare assistance, specifically supporting bed‐bound patients during feeding as shown in Figure 7A. These patients often experience limited upper limb mobility, making it difficult to reach food placed on a table beside the bed. In such cases, a co‐bot assists by retrieving food from the table and bringing it close to the patient's mouth. By maintaining the co‐bot's stiffness at a low level, the patient can easily adjust the position of the food with minimal force applied to the end‐effector, guiding it into their mouth with little effort. Importantly, the co‐bot's low stiffness minimizes the interaction force in the event of an unexpected obstacle along its path, ensuring that any collisions do not cause a significant impact or interrupt the food‐delivery process. This feature enhances the safety of the system, providing the patient with highly secure and reliable assistance during feeding.

To simulate this scenario, we created a representative feeding testing environment as shown in Figure 7B. The 3‐DoF RBBL configuration was strategically chosen based on a comprehensive kinematic trade‐off between workspace reachability, control simplicity, and interaction compliance. Fundamentally, the bedside feeding task represents a three‐dimensional spatial positioning challenge, requiring the end‐effector to navigate a 3D coordinate trajectory from a bedside platform to a patient's mouth. To govern this 3D position, a minimum of 3 degrees of freedom (3‐DoF) is strictly required. Considering the structural joint limits of individual modules, the RBB joint layout represents the most concise topology available within our modular framework that fully satisfies these spatial accessibility requirements. Utilising higher‐DoF assemblies (such as 4‐DoF topologies) would introduce redundant kinematic complexity and increase global system mass without offering functional advantages. Additionally, as verified by the workspace simulation envelopes, this specific modular stack yields a vertically extended volume that perfectly matches the bedside trajectory profile. By prioritising the flexible link (L) and a bending joint (B) at the distal end, the configuration explicitly concentrates the system's inherent mechanical compliance at the primary user‐interaction zone, ensuring that the patient can manually adjust the end‐effector with minimal guiding forces.

During the feeding sequence, the co‐bot follows a pre‐defined trajectory planned by the proposed control framework. Upon delivery, the patient holds a handle attached to the end‐effector and guides the food to their mouth. A force/torque (F/T) sensor integrated into the handle records the contact force throughout the process as shown in Figure 7D, indicating the amount of effort required by the patient to consume food with the co‐bot's assistance. Concurrently, the actuation pressure in each joint is monitored by the regulator's feedback system as shown in Figure 7C. To provide a rigorous statistical evaluation, the feeding experiment was executed across five independent repeated trials (*n* = 5), with the resulting pressure and multi‐axis force profiles plotted as the statistical Mean curve enclosed within a continuous Standard Deviation (± SD) shaded error band (Figure 7C,D). The re‐processed quantitative data show that the statistical maximum peak contact force during the food‐picking phase reaches 3.63 N along the Z‐axis, 2.42 N along the X‐axis, and 1.20 N along the Y‐axis. Once delivered, the patient manually waves the fork within a localized zone to guide the food. The recorded continuous force profiles across the X, Y, and Z trajectories demonstrate highly bounded tracking variations, maintaining minor steady‐state mean error drifts of only 0.14, 0.09, and 0.16 N, respectively. These extremely low force thresholds confirm that the co‐bot's low‐stiffness adaptation minimizes patient effort and maximizes collaborative comfort.

**FIGURE 8:**
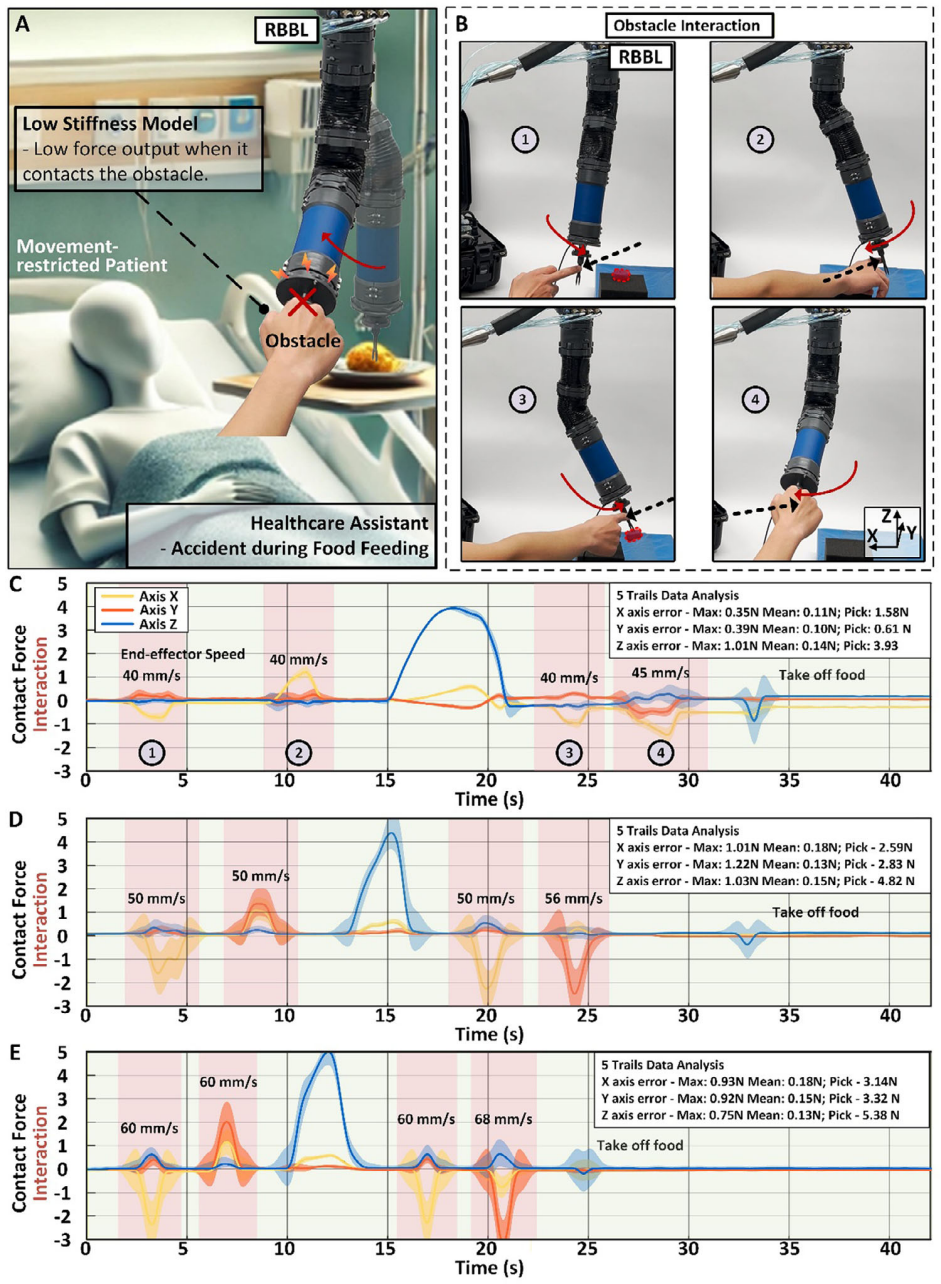
Multi‐velocity boundary interaction evaluations and collision safety compliance characterizations. (A) Operational schema of an accidental obstacle collision during close‐proximity clinical assistance. (B) Sequential physical snapshots capturing the physical interaction sequence (phases 1 to 4) between the moving 4‐DoF RBBL manipulator and a human arm. (C–E) Comprehensive multi‐axis interaction contact force response timelines evaluated under three distinct scaled operational velocity profiles: (C) 40 mm/s base speed, (D) 50 mm/s base speed, and (E) 60 mm/s base speed. All data are plotted as the statistical Mean across five trials (*n* = 5) seamlessly enveloped by a continuous Standard Deviation (± SD) shaded error band, with localized phase velocities and maximum multi‐axis peak impact thresholds explicitly annotated.

To further investigate system safety across a wider range of operating conditions, we simulated potential accidental collisions (as shown in Figure 8A) by manually introducing obstacles along the co‐bot's movement path at three distinct baseline execution velocities: 40 mm/s (Figure 8C), 50 mm/s (Figure 8D), and 60 mm/s (Figure 8E), with each velocity regime subjected to five independent repeated trials (*n* = 5). As shown in the physical sequence in Figure 8B and the consolidated multi‐axis timelines (Mean ± SD shaded bands) in Figure 8C–E, the robot's localized velocities and peak impact forces were explicitly mapped. The empirical results delineate a clear velocity‐dependent safety correlation: as the base operational speed scales up (triggering local acceleration peaks up to 68 mm/s during rapid segments), the recorded maximum contact forces predictably increase. Specifically, the peak interaction force during food retrieval increases from 3.93 N at 40 mm/s, to 4.82 N at 50 mm/s, and reaches 5.38 N under the 60 mm/s configuration, while the global mean tracking variations across all trials remain well below 0.18 N. Crucially, after the obstacles were removed, the food delivery process seamlessly continued and was completed without interruption. These tightly bounded contact force results confirm that the proposed co‐bot system does not generate dangerous impact forces or disrupt the operational flow even during high‐speed accidental collisions, mathematically proving the excellent passive compliance, repeatability, and intrinsic safety of the variable‐stiffness framework for sensitive healthcare environments.

### 2.3 Industrial Assistance Scenario: Co‐Bot RB for Chamfering Workpiece Holding

The second application scenario involves an industrial task—chamfering assistance—where tools are operated with both hands, and workpieces must be securely held in various orientations. This setup requires a support system that offers high stiffness and adjustable orientation. Our co‐bot addresses these requirements using a low DoF configuration that offers exceptional variable stiffness capabilities. To demonstrate this, we conducted a chamfering process where the co‐bot held a workpiece made from Though 2000 material resin, 3D‐printed for this purpose. This allowed us to manually operate a rasp while simultaneously adjusting the orientation of the workpiece.

To assess the co‐bot prototype's ability to effectively assist workers in completing the chamfering process, we recorded both the contacting force and the fluctuation of the workpiece using an F/T sensor and the NDI motion tracking system. We then inspected the chamfering quality of the workpiece by evaluating the parallelism and the lengths of the two legs in both chamfering triangles.

**FIGURE 9:**
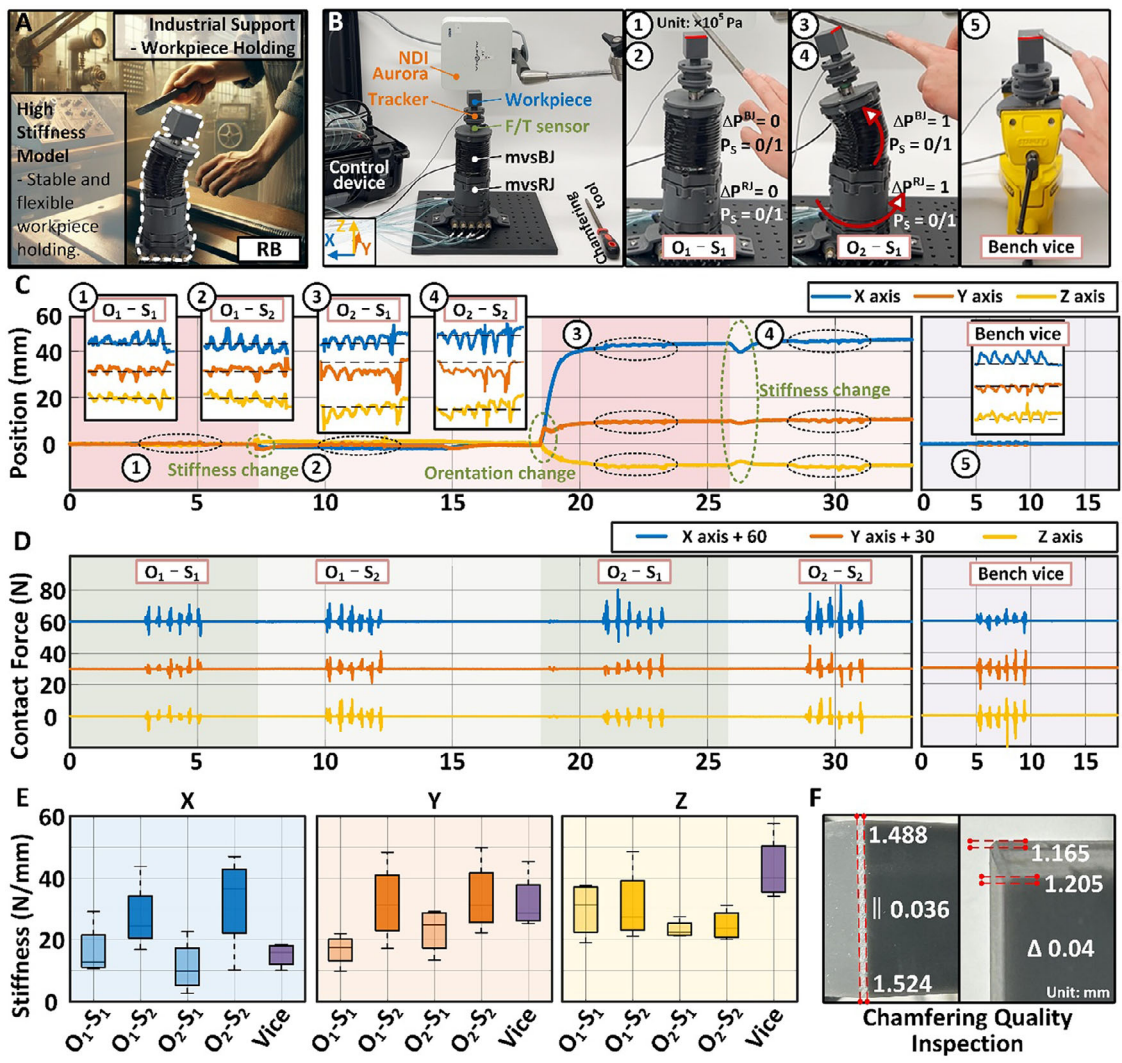
Results of Industrial Manufacturing Support. (A) The co‐bot with the RB configuration, setting as high stiffness model, holds a workpiece to support the worker to conduct the chamfering process. (B) The experimental setup for mimicking the support of the chamfering process, where the F/T sensor was mounted between the end of the mvsBJ and the holding mechanism of the workpiece, and the tracking sensor is mounted on the top of the F/T sensor to record the position change during the chamfering. In ① ②, there are two different stiffness models in the same stand position. In ③ ④, there are two different stiffness models in the same bending position. In ⑤, there is the same setup mounted on a traditional bench vice for comparison purposes. (C) The recording of the position change of the workpiece in 5 situations. (D) The recording of the force from the force sensor in 5 situations. (E) The stiffness result calculated by the recorded position change and force. (F) The quality inspection of the chamfer after we finished the chamfering process on the co‐bot.

As shown in Figure 9A, a co‐bot, specifically designed for tasks requiring high stiffness and orientation‐changing capabilities, constructed using an mvsBJ and an mvsRJ, is introduced to perform chamfering in this industrial application. The chamfering quality requirements include a leg length of 1 mm and a chamfering angle of 45 degrees. An F/T sensor is embedded between the workpiece and the co‐bot tip to detect the contacting force during the chamfering. Additionally, to monitor workpiece deflection during chamfering, an NDI Aurora tracker is integrated at the base of the workpiece.

A total of five manufacturing support scenarios were comprehensively evaluated under explicit pneumatic boundary conditions to assess the workpiece holding capability. The four co‐bot testing states represent an orthogonal combination of two discrete operational orientations (*O*_1_,*O*_2_) and two stiffness modes (*O*_1_ − *S*_1_,*O*_1_ − *S*_2_,*O*_2_ − *S*_1_, and *O*_2_ − *S*_2_). In the baseline orientation state (*O*_1_), the joint motion actuation pressures were maintained at Δ *P^BJ^* =  Δ *P^RJ^* =  0 bar (0 Pa). Under the *O*_1_ − *S*_1_condition, the stiffness‐regulating pressure was also set to *P_s_* =  0 bar, while under the *O*_1_ − *S*_2_condition, *P_s_* was pressurized to 1.0 bar (1.0 × 10^5^ Pa) to actively stiffen the joints through antagonistic fluidic equilibrium. Conversely, in the reoriented state (*O*_2_), the joint actuation profiles were incremented to a constant displacement pressure of Δ *P^BJ^* =  Δ *P^RJ^* =  1.0 barto rotate and bend the workpiece into the second working posture. The corresponding stiffness mode was evaluated at *P_s_* =  0 bar (for *O*_2_ − *S*_1_) and subsequently at *P_s_* =  1.0 bar (for *O*_2_ − *S*_2_). For comparison, the fifth benchmarking scenario utilized a conventional mechanical bench vice. The precise tracking deflection waveforms and multi‐axis contact force results during the chamfering cycles are systematically detailed in Figure 9B.

For comparison, the same workpiece was clamped in a bench vice, and the chamfering process was repeated while recording deflection and contact force. In each chamfering scenario, six chamfering motions were performed to complete the process. The workpiece position data is presented in Figure 9B, with X, Y, and Z values represented by blue, orange, and yellow curves over time. Detailed deflections for each chamfering action are shown in the five zoomed‐in regions. Contact forces in the X, Y, and Z directions are displayed separately in Figure 9C.

The co‐bot's stiffness in the X, Y, and Z axes during each chamfering action was calculated based on the measured contact forces and deflections, as summarized in Figure 4D,E. This data reflects the co‐bot's and bench vice's capabilities in securing the workpiece. According to the box plots, increasing the stiffness pressure P_s_ significantly improved the co‐bot's stiffness in the X and Y directions, reaching levels comparable to the bench vice when P_s_ = 1 in both orientations. In contrast, increasing P_s_ had little effect on stiffness in the Z direction for orientation one, as it primarily depends on the co‐bot's structural stiffness. However, in orientation two, Z‐axis stiffness nearly doubled with increased P_s_. Nonetheless, the bench vice maintained the highest stiffness along the Z axis. Notably, the bench vice exhibited lower stiffness in the X direction due to the round base of the workpiece, which hindered steady clamping in that axis. Overall, these results suggest that the co‐bot designed for chamfering tasks provides a similar workpiece fixation capability to the traditional bench vice.

Additionally, we conducted a chamfering quality inspection to evaluate the co‐bot's effectiveness in the process. An image of the chamfer was processed in MATLAB, where the Hough transform, algorithm was used to detect the chamfer edge, and the corresponding quality parameters were calculated. The parallelism of the chamfer was measured at 0.036 mm, with a leg length difference of 0.04 mm, resulting in a chamfering error of less than 4%. The results confirm that the co‐bot can maintain the necessary stiffness and positional stability, effectively supporting the completion of a precise and high‐quality chamfering operation.

## 3 Discussion

In this paper, we propose a modular and variable‐stiffness co‐robot system. Using the proposed design and fabrication process, we produced three mvsBJ, three mvsRJ, and two mvsL prototypes, incorporating ten internal actuation channels. Each prototype was then characterized. The mvsBJ prototype achieved 55.96 degrees of movement in each direction by controlling the actuation pressure ΔP_b_ and exhibited a threefold stiffness variation by controlling the pressure P_s_. The mvsL prototype showed a deflection of 6.72 mm with a length of 100 and 18.85 mm with a length of 150 mm. Additionally, the mvsRJ prototype achieved a 145‐degree rotational angle by adjusting the actuation pressure ΔP_r_. A control device was also developed to regulate the actuation pressure for the co‐bot system.

Next, all potential configurations constructed from the produced components were analyzed in a MATLAB simulation environment using the D‐H matrix. As a result, three specific configurations—RRRBL, RBRBL, and BRBBL—were selected for stiffness evaluation, confirming that pressure P_s_ effectively modulates the co‐bot's overall stiffness, although different configurations exhibit distinct characteristics.

Subsequently, an open‐loop control framework was developed, accounting for configuration, load, self‐gravity, and stiffness, to control the end‐effector's trajectory using the MATLAB Robotics System Toolbox. The three selected co‐bot configurations were actuated to follow both circular and straight trajectories. Crucially, the global statistical repeatability, geometric boundary compliance, and operational robustness of the reconfigurable co‐bot platform were comprehensively synthesized across five independent experimental trials (*n* = 5) under varied dynamic constraints. As systematically demonstrated by the coupled multi‐axis tracking data, the proposed pressure‐dependent compensation control framework introduces exceptional inter‐trial stability. The statistical Mean trajectories remain tightly enveloped within continuous Standard Deviation (± SD) shaded error bands, successfully restricting steady‐state position drifts and mean error variations to a localized precision threshold of less than 0.16 N in force feedback and tight spatial coordinates during user‐guided tracking.

Concurrently, evaluation of the absolute geometric error distributions confirms that the system maintains robust path tracking predictability even when subjected to severe external disturbances. Under a heavy 500 g terminal payload, the vertical Z‐axis deviations of the 3‐DoF BRBBL configuration are strictly bounded within ± 2 mm, while the interquartile spatial error tracking bands for the complex 4‐DoF RRRBL circular loop maneuvers remain securely locked within ± 4 mm.

Beyond baseline kinematic accuracy, the statistical synthesis of the healthcare application scenarios delineates a critical, velocity‐dependent physical safety law. As the baseline execution velocity scales up across three distinct testing regimes (40, 50, and 60 mm/s), introducing dynamic transient acceleration spikes up to 68 mm/s, the peak interaction contact forces safely and predictably scale from 3.93 N to a maximum of 5.38 N. Crucially, the low standard deviations and tightly constrained variance profiles across these high‐speed accidental collision boundaries mathematically prove that the embedded antagonistic fluidic muscle networks provide reliable passive deceleration and instant energy absorption. This structural‐control compliance coupling successfully buffers physical human‐robot impacts well below established pain thresholds, establishing a balanced, transparent validation roadmap for the system's applicability in safe, dynamic human‐collaborative automation.

To further contextualize the scientific contributions of this work, a critical comparison with existing state‐of‐the‐art variable‐stiffness robotic systems is warranted. Conventional continuous or soft robotic structures (e.g., those utilizing particulate jamming, layer jamming, or antagonistic fluidic‐tendon architectures) excel in yielding exceptionally high maximum structural rigidity or high dynamic tracking bandwidth via closed‐loop feedback. However, these systems often sacrifice modular adaptability and topology reconfigurability, as their variable‐stiffness mechanisms are heavily integrated into a monolithic trunk.

The proposed modular and variable‐stiffness co‐robot framework uniquely overcomes this limitation by decoupling architectural topology from mechanical control. By isolating joint bending (mvsBJ), joint rotation (mvsRJ), and variable‐length rigid linkages (mvsL) into distinct, reconfigurable modules, users can rapidly assemble customized co‐bots (such as the RRRBL, BRBBL, and RBRBL configurations evaluated herein) tailored to explicit task geometries. Furthermore, the embedded pressure‐dependent compensation control algorithm can mathematically counteract structural deflections induced by self‐weight and external loads without relying on dense sensory arrays.

Nonetheless, certain operational limitations must be noted. Unlike jamming mechanisms that exhibit rapid, step‐like stiffness transitions, the pneumatic pressure regulation utilized in our prototypes requires steady‐state convergence. More importantly, the current control framework operates in an open‐loop fashion. This implies that highly non‐linear soft material traits—such as silicone‐layer viscoelastic hysteresis, long‐term polymer fatigue, and dynamic transient oscillations—remain uncompensated outside the analytical kinematic loop. Consequently, while our architecture guarantees customizable flexibility and inherently safe physical interactions under steady‐state operational boundaries, its application remains primarily suited for quasi‐static or steady‐state human‐robot collaborative tasks.

## 4 Conclusion

This paper presented a modular and variable‐stiffness co‐robot (co‐bot) system designed to enhance adaptability, safety, and task versatility in human–robot collaboration scenarios. A systematic design and fabrication framework enabled the development of multiple modular variable‐stiffness components (mvsBJ, mvsRJ, and mvsL), integrating ten internal actuation channels. Experimental characterization demonstrated large motion ranges and significant stiffness modulation capabilities, confirming the effectiveness of pressure‐based actuation and stiffness regulation.

Comprehensive configuration analysis using D–H modeling in MATLAB identified feasible multi‐module assemblies and revealed that stiffness pressure effectively regulates the overall structural stiffness, while different configurations exhibit distinct mechanical characteristics. An open‐loop control framework incorporating configuration, load, self‐gravity, and stiffness effects was further developed and validated through trajectory tracking experiments. The results demonstrated satisfactory tracking performance for both circular and linear paths, confirming the feasibility of the proposed modeling and control strategy.

Two representative application scenarios—healthcare assistance and industrial task support—further highlighted the system's versatility. The feeding assistance task demonstrated safe human interaction with minimal required user force and low collision forces, validating the intrinsic compliance and safety of the system. Conversely, the chamfering task showcased the co‐bot's ability to achieve high stiffness when required, enabling stable object fixation comparable to conventional mechanical fixtures.

Overall, the proposed co‐bot system successfully integrates modularity, variable stiffness control, and adaptable configuration design within a unified framework. The results demonstrate its strong potential for deployment in diverse human‐centred environments where both safety and task‐dependent rigidity are essential. However, despite these validated advantages, several critical technical challenges remain to be addressed, bounding the current claims within quasi‐static operational environments. First, the control framework operates in an open‐loop fashion, leaving the system vulnerable to cumulative positioning drift caused by the inherent non‐linear properties of soft silicone components. Second, complex structural non‐linearities—such as viscoelastic hysteresis, multi‐channel pneumatic latency, and long‐term elastomeric fatigue under cyclic loading—are not yet dynamically compensated.

To overcome these limitations and provide a clear roadmap for the future development of this platform, our upcoming research will prioritize three strategic pillars: (1) Sensory Closed‐Loop Control: integrating flexible tactile arrays or embedded electromagnetic tracking sensors directly into the modules to achieve real‐time positional closed‐loop feedback; (2) Dynamic Compensation Modeling: developing advanced viscoelastic‐viscoplastic models to actively predict and suppress transient structural oscillations and hysteresis during high‐velocity maneuvers; and (3) System‐Level Scalability: transitioning from stationary desktop workstations to integrating these customizable soft‐rigid configurations onto mobile robotic bases to evaluate their adaptive interaction limits across broader medical assistance and dynamic industrial contexts. Addressing these bottlenecks will transition the proposed framework from a customizable laboratory prototype into an autonomous, robustly adaptive co‐bot platform.

## 5 Methods

### 5.1 The Co‐Bot Components Construction

The soft components of three types of co‐bot modules, namely, the silicone chamber of the mvsBJ, the silicone cylinder of the mvsL, and the bellow chamber of the mvsRJ, were first analyzed using finite element analysis (FEA) simulations to determine their optimal design parameters.

**FIGURE 10:**
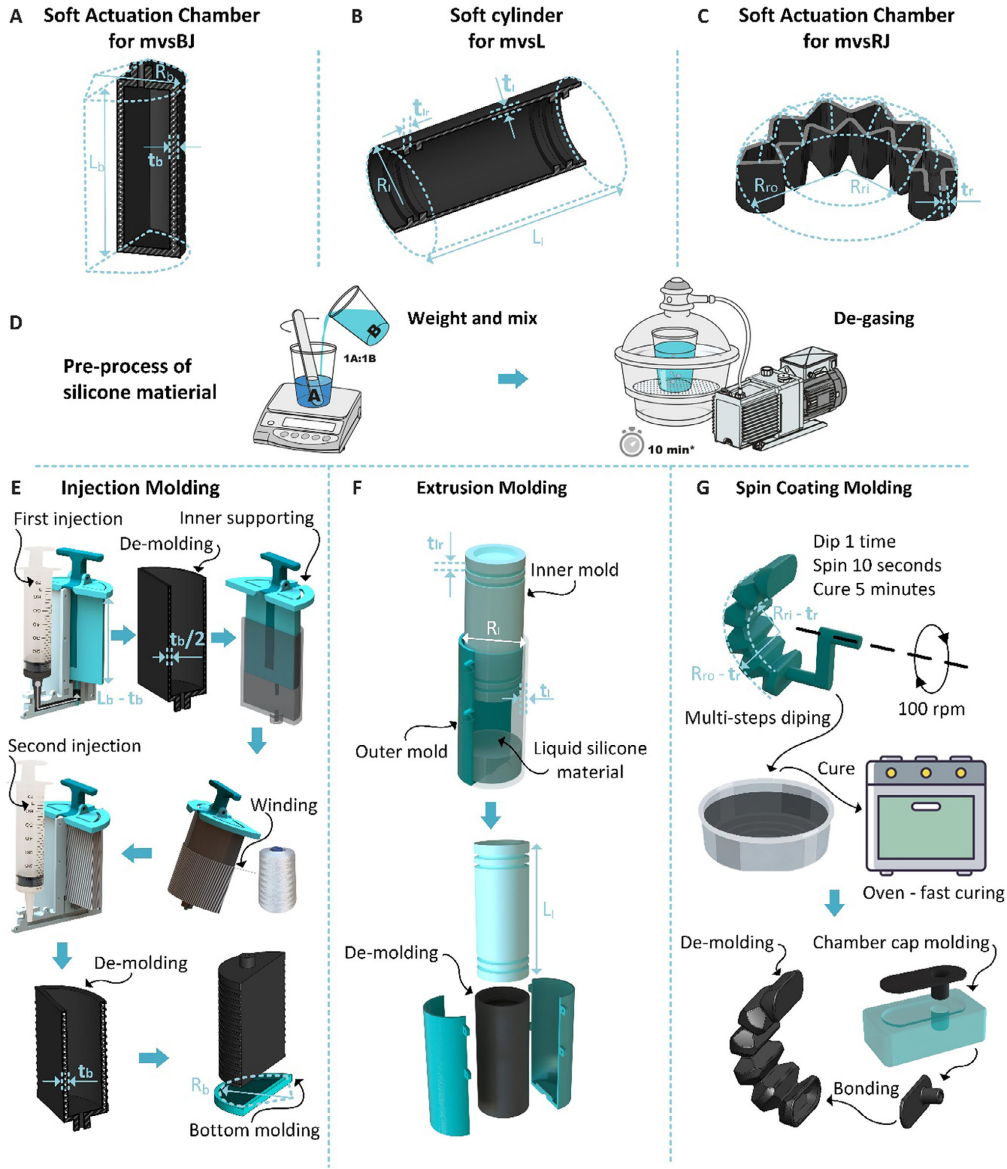
The fabrication process of three key soft parts in each co‐bot components. (A) The structure indication of the mvsBJ soft actuation chamber. (B) The structure indication of the mvsL soft cylinder. (C) The structure indication of the mvsRJ soft actuation chamber. (D) The common pre‐process of the silicone material. (E) The injection molding process of mvsBJ actuation chamber. (F) The extrusion molding process of mvsL cylinder. (G) The spin coating molding process of mvsRJ actuation chamber.

Subsequently, the key soft parts of the three module types were fabricated using a diverse silicone molding process, while the rigid components were 3D printed. Figure 10A–C illustrates the structures of these three distinct soft components. Figure 10D presents the common pre‐processing steps for the silicone material, including mixing and de‐gassing. Figure 10E details the injection molding process for the mvsBJ actuation chamber, which involves two steps. In the first step, a chamber wall with half of the final thickness is formed. A reinforcing thread is then wound around the surface of this partially formed wall. In the second step, the remaining half of the wall is molded, followed by the sealing of the chamber base using a bottom mold to complete the fabrication process. Figure 10F shows the extrusion molding process for the mvsL cylinder. The cylinder's shape is formed directly by the cavity between the inner and outer molds.

Given the structural complexity of the mvsRJ chamber, a spin‐coating process was adopted, as shown in Figure 10G. The inner cavity of the chamber is 3D printed to serve as the mold, which is repeatedly dipped into silicone rubber liquid until the desired wall thickness is achieved. After each dip, the mold is mounted on a spin mechanism and rotated at 100 rpm for 10 s to ensure uniform material distribution. The coated mold is then cured in an oven set at 60°C. Each dip‐and‐cure cycle adds approximately 0.2 mm to the wall thickness. Once the desired thickness is reached, the cured silicone layer is demolded, and a cap mold is used to form the chamber's top section, completing the fabrication process.

### 5.2 Choice of Key Soft Components Material Choose and Determination of Design Parameter

**FIGURE 11:**
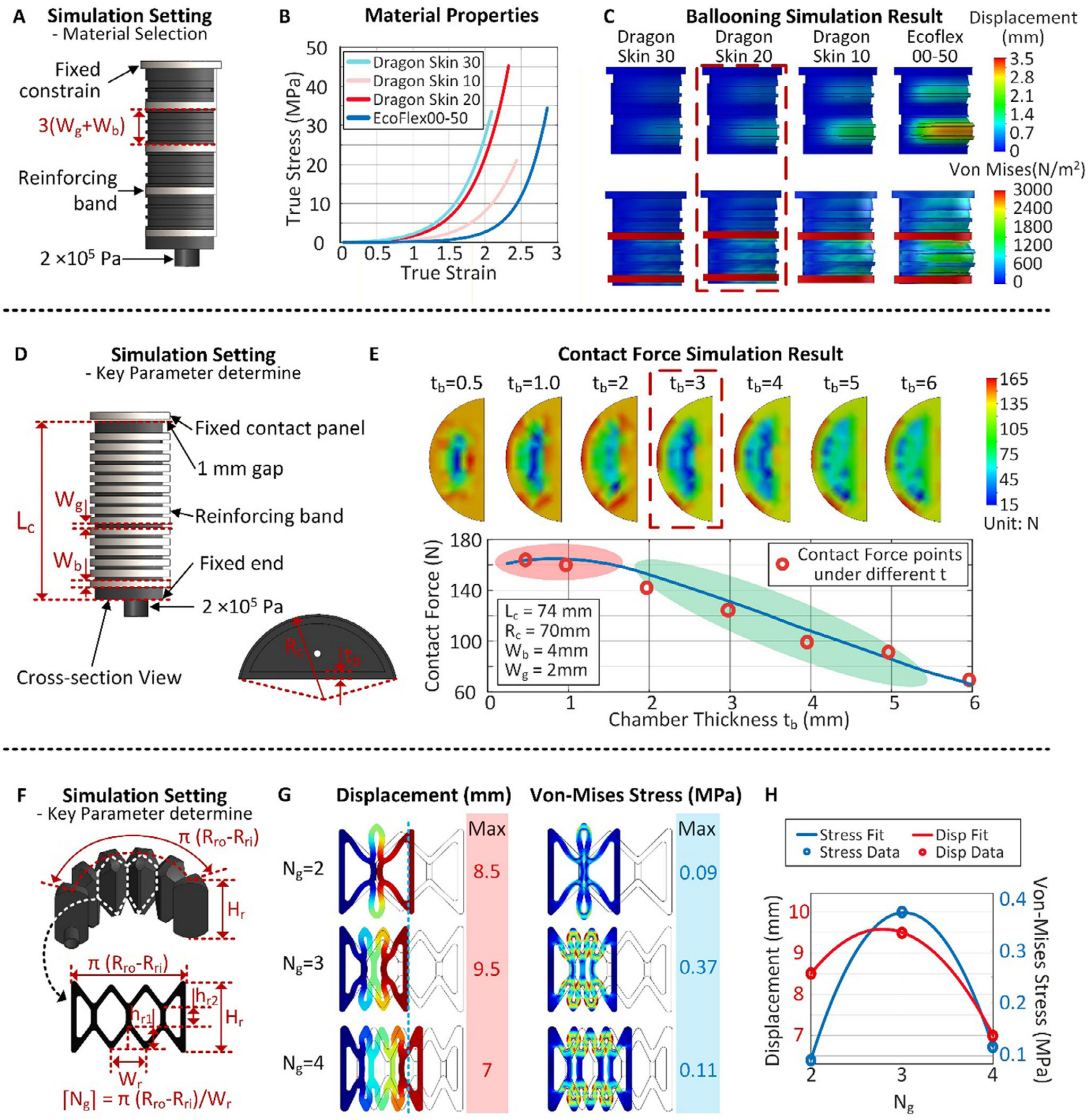
FEA‐based design analysis of key soft component parameters. (A) FEA simulation configuration for localized wall ballooning evaluations under an inflation pressure of 2 × 10^5^ Pa, using an exaggerated gap distance metric defined by 3(Wg + Wb). (B) Uniaxial tensile true stress–strain characterization profile curves for the four candidate silicone rubbers (Dragon Skin 30, Dragon Skin 20, Dragon Skin 10, and EcoFlex 00–50). (C) Coupled ballooning simulation results comparing spatial displacement distributions (top row) and corresponding internal von Mises stress fields (bottom row) across the evaluated materials, quantitatively identifying Dragon Skin 20 as the optimal substrate. (D) Cross‐sectional simulation setup and geometric parameter definition layout for investigating contact force optimization as a function of chamber wall thickness (tb). (E) Contact force simulation results, featuring localized stress distribution nephograms under varied thickness boundaries (from tb = 0.5 mm to tb = 6 mm) alongside the corresponding quantitative contact force attenuation curve. (F) Geometric schema, boundary settings, and dimensional parameter abstractions for the multi‐convolution bellows configuration within the mvsRJ module. (G) Detailed multi‐segment FEA deformation profiles under axial compression, comparatively delineating the localized displacement fields (left column) and von Mises stress concentration zones (right column) across distinct convolution metrics (Ng = 2, 3, and 4). (H) Integrated multi‐axis optimization curves plotting maximum displacement output and internal von Mises stress profiles as a function of the bellows convolution number (Ng), establishing the critical mechanical trade‐off at Ng = 3.

To select appropriate silicone rubber materials for the key soft components of different modules, we employed finite element analysis (FEA) simulations. When the chamber is inflated, its wall stretches, causing the gap between successive reinforcing bands to widen. This widening can lead to localized ballooning of the wall, which in turn increases the risk of failure and material damage. Our observations indicate that softer materials exhibit more pronounced ballooning behavior. To address this, we constructed an FEA model of the actuation chamber in the mvsBJ module to evaluate and compare the performance of different silicone rubbers. The simulation setup is illustrated in Figure 11A, where W_b_ represents the ribbon width of the fiber bands used to construct the wrapped fiber‐reinforcement layer, and W_g_ denotes the pitch distance between successive loops during fiber wrapping. We used a gap distance three times larger than the standard value (3(W_g_ + W_b_)) to mimic the chamber's extension under actuation. Four types of silicone rubber—Dragon Skin 30, Dragon Skin 20, Dragon Skin 10, and Ecoflex 50 – were evaluated, each with distinct stiffness properties.

Their true stress–strain curves, obtained from uniaxial tensile tests, are shown in Figure 11B. To systematically justify the mechanical integrity of this material selection, the specific boundary conditions and internal stress implications of the FEA model were thoroughly evaluated. The simulation domain was configured by applying a rigid fixed constraint to the top flange of the actuator module, while defining a uniform internal pneumatic pressure loading of 2 × 10^5^ Pa along the fluid‐structure interface. The resulting stress profiles in Figure 11C demonstrate that while stiffer materials successfully suppress the peak ballooning displacement from 3.5 mm (Ecoflex 00–50) down to less than 0.7 mm (Dragon Skin 30), they introduce significant strain localization. Specifically, the maximum internal von Mises stress severely scales up, exceeding a peak of 3000 N/m^2^ clustered precisely around the fiber‐reinforcement anchoring boundaries for Dragon Skin 30. Evaluating these coupled geometric deflections and material stress fields ensures that the chosen silicone candidates maintain safe mechanical margins during operation.

In addition to material selection, the wall thickness of the soft components is another critical design parameter. We conducted a second set of simulations to examine how wall thickness affects the output force of the chamber, as shown in Figure 11D. Simulation results (Figure 11E) reveal a nearly linear increase in output force with decreasing wall thickness, up to a threshold. When the wall thickness drops below 2 mm, the rate of force increase diminishes, indicating a trade‐off region. The green‐shaded zone in Figure 11E represents the optimal range for adjusting output force. However, minimizing wall thickness too aggressively could exacerbate the ballooning issue discussed earlier. Therefore, an appropriate balance must be struck between achieving high output force and maintaining structural integrity.

Together, these two sets of simulation results provide a solid foundation for selecting suitable material types and determining the optimal wall thickness for the soft components in both actuation chambers.

For the mvsRJ module, which features a bellows structure, another important design parameter is the number of bellows convolutions, denoted as Ng. This number influences the minimum compressed height of the chamber, which in turn affects the module's maximum achievable rotation angle. We used simulations to explore how different values of N_g_ affect this compressed length. As illustrated in Figure 11F, the inner height h_r2_ is constrained by the maximum elongation ratio of the chosen material, while the outer height h_r1_ is determined by the total chamber height H_r_. Given a selected bellows width W_r_, N_g_ can then be calculated based on the chamber's radial dimensions. FEA simulations were conducted for various values of N_g_. The displacement mesh generation for the multi‐convolution bellows was subjected to an axial compressive loading, with the simulation termination criterion strictly governed by the non‐linear geometric self‐contact between adjacent internal surfaces. Results shown in Figure 11G, including the corresponding maximum displacement and von Mises stress. As plotted in Figure 11H, the optimal displacement is achieved when N_g_ is set to 3. When N_g_ further increases to 4, the displacement drops because the simulation termination criterion is governed by structural self‐contact; the higher number of convolutions causes the thickness of the internal silicone walls to accumulate within the fixed axial space, leading to premature physical contact between neighboring inner surfaces that halts further compression. However, this N_g_ = 3 configuration also generates the highest internal stress during compression, highlighting a critical trade‐off between deformation capability and mechanical robustness.

### 5.3 Analytical Actuation Model for Three Different Modules and Control Framework for Co‐Bot With Different Configurations

#### 5.3.1 Actuation Model for mvsBJ

**FIGURE 12:**
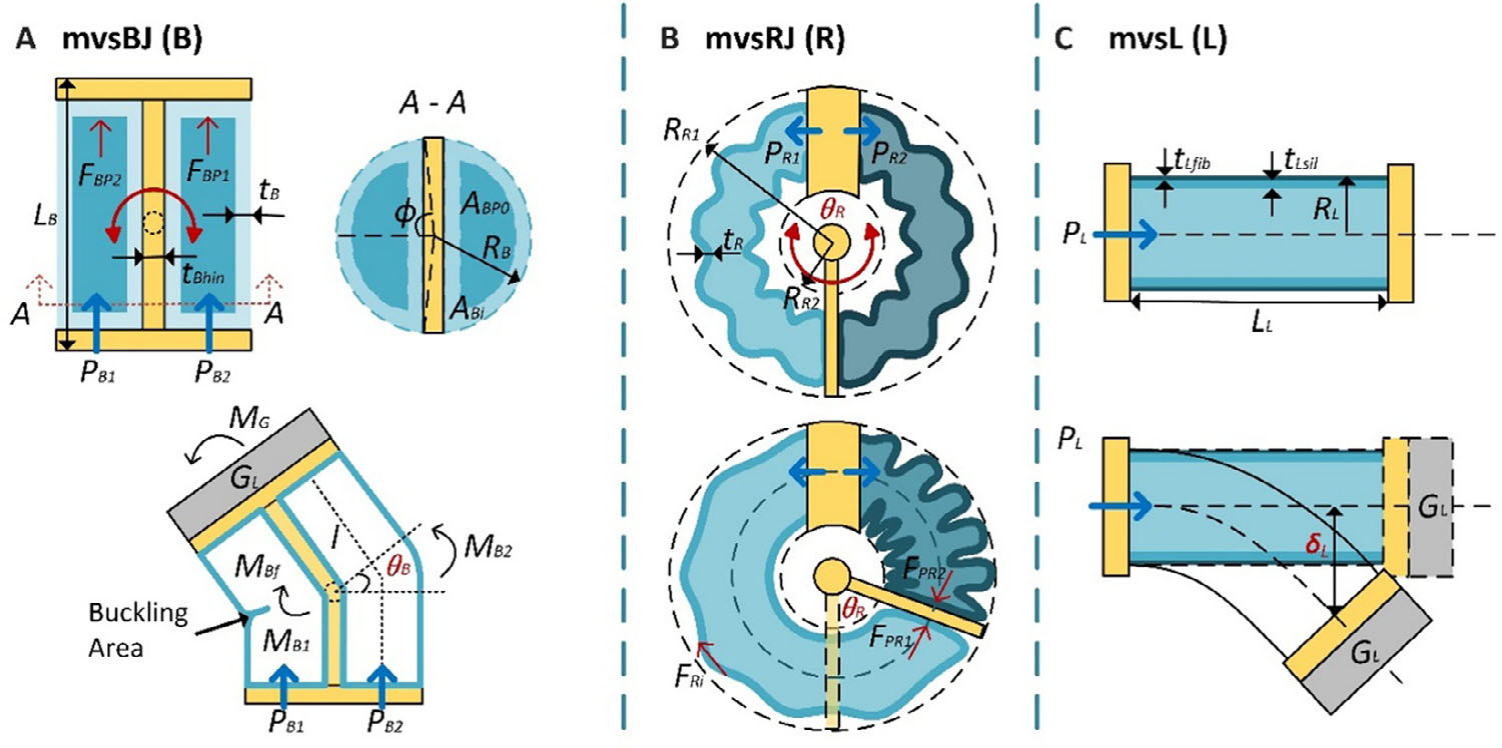
Analytical actuation models and structural schematics of the three modular soft actuation units. (A) Schematic and modeling of the module mvsBJ. The mvsBJ consists of two internal chambers actuated antagonistically. A pressure difference Δ*P_B_* leads to asymmetric chamber deformation and results in a bending angle θ_*B*_. The extending chamber is reinforced with embedded fibers, producing primarily axial strain, modeled using the Mooney‐Rivlin hyperelastic formulation. Resulting torques are computed from pressure and resistive forces, and a mapping function $f_{\theta }^{B}\left(\Delta P_{B}\right)$ is derived under quasi‐static equilibrium. (B) Schematic and force model of mvsRJ. This module shares similar material and chamber structure with mvsBJ but features a rotational output. The actuation forces from chambers with pressures *P*_*R*1_ and *P*_*R*2_ are balanced with resisting stress and a simplified constant frictional term. The rotational angle θ_*R*_ is then mapped to pressure difference via $f_{\theta }^{R}\left(\Delta P_{R}\right)$. (C) Analytical model of the module mvsL, modeled as a fiber‐constrained pressure‐tunable cantilever beam. Internal pressure *P_L_* increases the effective Young's modulus (*E_Leff_*), enabling modulation of beam stiffness. Based on Euler–Bernoulli beam theory, the end deflection δ_*L*_ under an external load *G_L_* is expressed as a function of internal pressure.

To numerically capture the relationship between actuation pressure and bending angle θ_*B*_—we develop a physics‐based model of the soft joint based on its geometry and material properties. As illustrated in Figure 12A, the soft joint consists of two internal chambers. When the pressure difference Δ*P_B_*  =  *P*_*B*2_ − *P*_*B*1_ is applied (*P*_*B*2_ > *P*_*B*1_), the joint bends anticlockwise with a bending angle θ_*B*_. The expansion of chamber 2 is constrained radially by embedded fiber reinforcements, resulting in primarily axial deformation. For simplicity, the entire chamber is assumed to be composed of silicone rubber, modeled using the Mooney‐Rivlin hyperplastic formulation:

$$\sigma_{B}=2\left({\lambda_{B}}^{2}-1/\lambda_{B}\right)\left[C_{10}+C_{01}/\lambda_{B}+2C_{20}\left({\lambda_{B}}^{2}+2/\lambda_{B}\;-\;3\right)\right]$$

where λ_*B*_ is the axial stretch ratio, and *C*_10_, *C*_01_, and *C*_20_ are material constants. The axial stress force in the extending chamber is *F_Bi_*  =  *A_Bi_* σ_*B*_ and the pressure‐generated force is *F*_*BP*2_  =  *A*_*BP*0_ *P*_*B*2_. The resulting torque about the bending axis is:

$$M_{B2}=\left(L_{B}/2\right)\left(F_{BP2}-F_{Bi}\right)$$

An external load applied at the joint generates an additional torque:

$$M_{BG}=\left(L_{B}/2\right)G_{L}sin\left(\theta_{B}\right)$$

Chamber 1 undergoes folding, leading to two resistive torques: *M_Bf_* (from the folding) and *M*_*B*1_ (from internal pressure). Based on a modified pressure‐tuned Euler–Bernoulli Beam Theory, they are approximated as:

$$M_{Bf}=-\frac{E_{Beff}I_{B}}{L_{B}}.\theta_{B}$$

$$M_{B1}=P_{B1}\;A_{BP0}\left(L_{B}/2\right)$$

where, the

$$I_{B}=\int_{-\phi }^{\phi }\int_{R_{B}-t_{Bhin/2}}^{R_{B}}r^{3}\sin\theta^{2}drd\theta$$

$$E_{Beff}=E_{B0}+\alpha_{B}P_{B1}$$

Assuming quasi‐static equilibrium, we have:

$$M_{B2}+M_{G}=M_{Bf}+M_{B1}$$

From this, a mapping function $f_{\theta }^{B}\left(\Delta P_{B}\right)$ is derived to express the steady‐state bending angle as a function of the pressure difference. This mapping serves as the basis for joint‐level pressure‐angle control and parameter optimization in the full co‐robotic system.

#### 5.3.2 Actuation Model for mvsRJ

Since the mvsRJ module employs the same silicone material and similar structure of the actuation chamber as the mvsBJ, we adopt a similar modeling approach with appropriate simplifications. The actuation forces generated by chamber 1 and chamber 2, as shown in Figure 12B, under internal pressures *P*_*R*1_​ and *P*_*R*2_ respectively, can be expressed as:

$$F_{RP1}=A_{RP0}\;P_{R1},\;\;F_{RP2}=A_{RP0}\;P_{R2}$$

The circumferential stress in the extending chamber contributes an opposing force:

$$F_{Ri}=A_{Ri}\;\sigma_{R}$$

Due to the bellows geometry of the chamber, the resistance force during compression is significantly reduced, as confirmed in previous simulations. Additionally, friction within the actuation cavity can be further minimized by applying lubricants. Therefore, the combined resisting force, accounting for friction and compression, is approximated by a constant term *f_R_*.

It is important to note that external loads applied on the central rotational shaft do not influence the circumferential force balance and can thus be neglected in this model. Under quasi‐static conditions, force equilibrium at the rotating plate between the two chambers yields:

$$F_{PR1}=F_{PR2}+f_{R}+F_{Ri}$$

It's easy to know that the σ_*R*_ associates the rotational angle θ_*R*_ from the simplified geometry. Thus, we could obtain the mapping function $f_{\theta }^{R}\left(\Delta P_{R}\right)$ from the above equations.

#### 5.3.3 Actuation Model for mvsL

To characterize the bending behavior of the soft pneumatic beam under an external end load *G_L_*, we consider a simplified analytical model based on the classical Euler–Bernoulli beam theory, modified to incorporate pressure‐dependent stiffness. The mvsL is also fabricated from the same silicone material as the mvsBJ and mvsRJ, and its outer surface is tightly wrapped with a non‐extensible fiber mesh, which prevents both axial elongation and radial expansion during internal pressurization. This constraint enables a geometric simplification, allowing the structure to retain a constant cross‐sectional profile while exhibiting variable stiffness as a function of internal pressure. We model the link as a pressure‐tunable cantilever beam with the following geometry shown in Figure 12C.

The second moment of area *I_L_* of the hollow circular cross‐section is given by:

$$I_{L}=\frac{\pi }{4}\;\left[R_{L}^{4}-{\left(R_{L}-t_{L}\right)}^{4}\right]$$

Due to the fiber constraint, the effect of internal pressure is assumed to increase the effective Young's modulus *E_Leff_* ​ of the structure without altering its geometry. Accordingly, the maximum deflection δ_*L*_ ​ at the free end under the load *G_L_* is given by:

$$\delta_{L}=\frac{G_{L}L_{L}^{3}}{3E_{Leff}\left(P_{L}\right)I_{L}}$$

The pressure‐dependent effective modulus *E_Leff_* (*P_L_*) is determined empirically by fitting to experimental data obtained under varying internal pressures. In the absence of experimental data, *E_Leff_* ​ may be approximated as:

$$E_{Leff}\;\left(P_{L}\right)=E_{L0}+\alpha_{L}P_{L}$$

where *E*_*L*0_​ is the baseline modulus of the unpressurized elastomer‐fiber composite, and α_*L*_ is an empirically determined pressure‐sensitivity coefficient. This formulation allows for rapid estimation of bending deflection under combined mechanical and pneumatic actuation and supports the design and control of soft robotic elements with tunable stiffness characteristics.

To systematically substantiate the predictive fidelity and accuracy of the developed analytical actuation models, rigorous experimental validations were executed across all three module archetypes, with quantitative error metrics directly annotated within the characterization profiles. For the mvsBJ bending module (Figure 2C), the analytical model exhibits an exceptional fit against the measured real bending angles under varying pressure differentials (ΔP_b_), yielding a Root Mean Square Error (RMSE) of 1.152 degrees, a Mean Absolute Error (MAE) of 0.885 degrees, a Maximum Error of 2.828 degrees, and a Coefficient of Determination (R^2^) of 0.9947. For the mvsL elongation module, the structural model captures the complex tip deflections (δ) across a wide tip loading spectrum (0 to 16 N) under distinct internal pressures. As delineated in Figure 3C (L_l1_ = 150 mm) and Figure 3D (L_l2_ = 100 mm), the framework maintains tight tracking boundaries, achieving RMSE values well below 1.33 mm and R^2^ metrics tightly locked between 0.957 and 0.997 across all active pressure thresholds. Furthermore, the rotational actuation profile for the mvsRJ module (Figure 4B) maps the analytical predictions against the empirical multi‐channel pressure spaces (P_r1_ and P_r2_), successfully achieving a global RMSE of 3.34 degrees and an R^2^ of 0.982 up to a maximum rotation angle (θ_r_) of 72.42 degrees. These tightly bound error profiles mathematically confirm that the proposed analytical models possess excellent predictive capabilities and robustness, providing a reliable kinematic foundation for the downstream closed‐loop and open‐loop trajectory control tasks.

Building on the proposed actuation models, we established a control framework for co‐bots with various configurations and developed a control device to facilitate precise motion control. The model‐based control methodology is thoroughly explained in Section S1.

### 5.4 The Co‐Bots Configuration Analysis

Based on the proposed co‐bot system prototype, the maximum number of internal actuation channels is 10. Considering that some channels are allocated to the end‐effector, the maximum number of joints (i.e., mvsBJ and mvsRJ) that can be incorporated into a co‐bot configuration is 4. Consequently, there are 16 potential co‐bot configurations, each exhibiting unique characteristics in terms of workspace and variable stiffness behavior. To evaluate these features, a comprehensive analysis of the workspace for all possible configurations was conducted, as detailed in Section S2. Building upon the workspace analysis, several representative configurations were selected for further investigation into their stiffness variation behavior. This analysis, presented in Section S3, assesses the capability of the proposed co‐bot system to achieve variable stiffness, providing insights into its performance and adaptability.

### 5.5 Trajectory Control of Typical Co‐Bots

**FIGURE 13:**
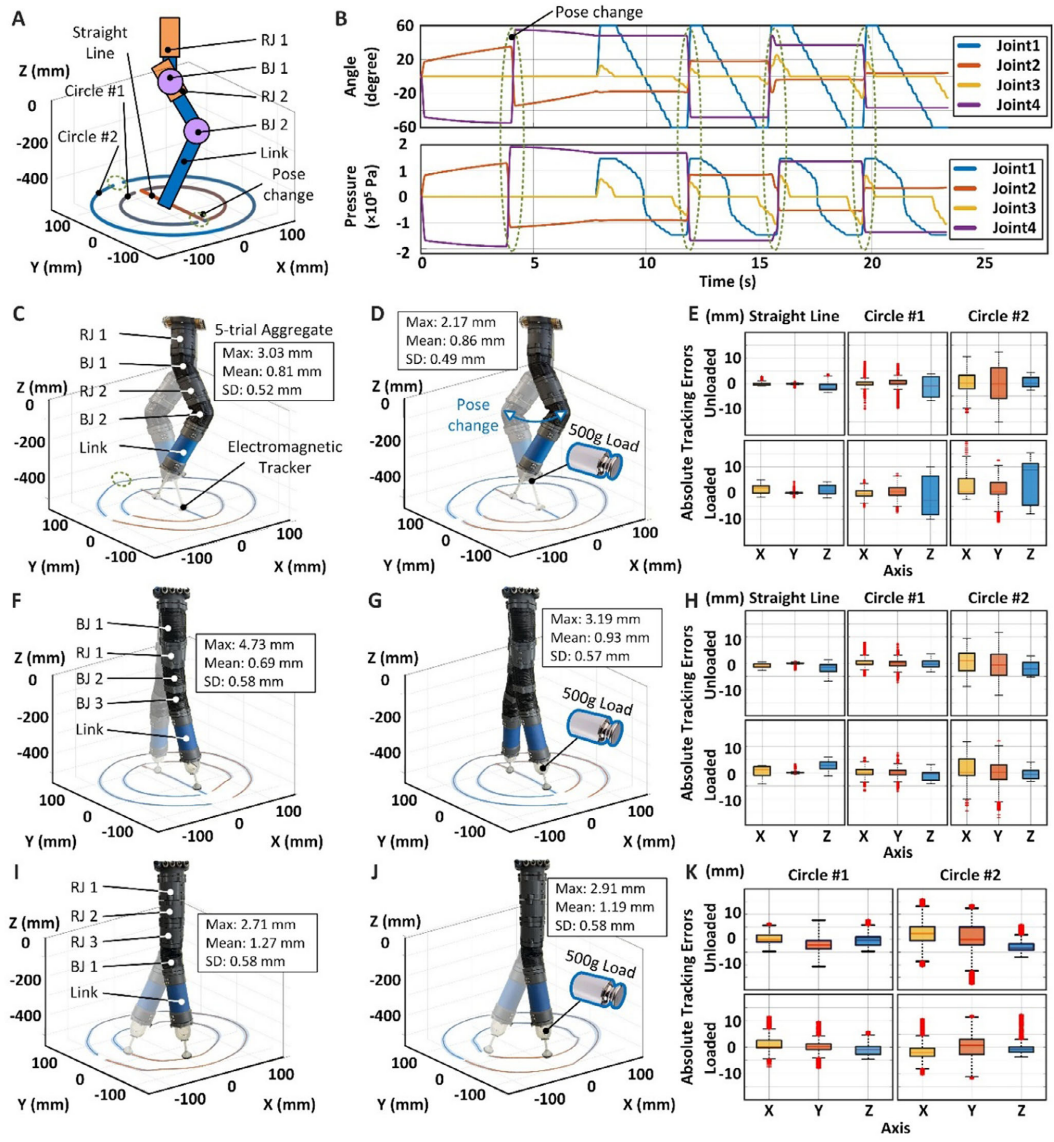
Statistical trajectory tracking control performance and repeatability characterizations across typical co‐bot configurations. (A) Spatial schema and geometric coordinates of the predefined reference paths, including the 3D straight line, Circle 1, and Circle 2, highlighting the automated pose change transitions. (B) Real‐time joint angle profiles (top) and corresponding internal multi‐channel pneumatic actuation pressure regulations (bottom) over the complete execution timeline. (C–E) Experimental validation for the 4‐DoF RBRBL configuration: (C) 3D trajectory tracking performance under the unloaded condition displayed as a 5‐trial statistical mean enclosed within a shaded standard deviation (± SD) error band, with localized structural component annotations and quantitative tracking error indicators; (D) 3D trajectory tracking profiles under a 500 g load boundary showing smooth tracking retention during pose changes; (E) Comparative box plots tracking the multi‐axis (X, Y, and Z) absolute tracking error distributions relative to the ground‐truth geometric paths across all trajectory shapes. (F–H) Experimental tracking characterization for the 3‐DoF BRBBL configuration: (F) Unloaded 3D end‐effector tracking trajectories over 5 repeated trials with labeled structural boundaries and localized Max, Mean, and SD error statistics; (G) Loaded 3D execution path behavior under a 500 g end‐effector load boundary; (H) Distributed multi‐axis absolute tracking error box plots under both unloaded and 500 g loaded environments. (I–K) Trajectory tracking validation for the 4‐DoF RRRBL configuration: (I) Unloaded multi‐circle (Circle 1 and Circle 2) 3D statistical tracking trajectories with corresponding spatial annotations; (J) 5‐trial aggregate 3D trajectory profiles operating under a 500 g task load; (K) Comprehensive multi‐axis absolute tracking error distributions displaying tight variance boundaries across the execution configurations.

After establishing the complete control framework in the previous section, a series of physical experiments were conducted to validate its feasibility. In these experiments, three selected co‐bot configurations, namely RRRBL, BRBBL, and RBRBL, were tested to follow three distinct trajectories: a straight line, a small circle, and a large circle. Using the proposed control framework, the actuation angles for each component were calculated within the MATLAB simulation environment by solving the inverse kinematics. An example of the simulated motion planning for the RBRBL configuration is shown in Figure 13A, where several gaps caused by pose changes are evident. Figure 13B further illustrates the actuation angles and corresponding pressures for each component over time.

To measure the end‐effector's position during the experiments, an NDI tracking system was utilized, as in previous setups. Additionally, a 500‐gram standard weight was attached to the end‐effector to simulate loading conditions, which serves as a representative external disturbance sufficient to validate the predictive compensation capability of the analytical kinematic control model under load variance. The results of the unloaded trajectory control for the three configurations are shown in Figure 13C,F,I, respectively. During these tests, significant pose changes were observed in certain regions, leading to gaps in the trajectories, as highlighted by green circles. For the loaded condition, with the 500‐gram weight applied, the trajectory control results are presented in Figure 13D,G,J. The recorded trajectory errors relative to the desired paths, in terms of the x, y, and z axes, are depicted in Figure 13E,H,K, with errors for the straight‐line and circular trajectories separated into individual error boxes.

The results demonstrate that all three configurations effectively followed the prescribed trajectories under the proposed control framework. The RBRBL configuration exhibited high precision during the straight‐line trajectory in both the unloaded and loaded scenarios, with the tracking error medians across all three axes remaining close to 0.2 mm and bounded within a narrow range of ± 3 mm (Figure 13E). However, during the circular trajectory (Circle #1), the multi‐axis coupling effects amplified the deviations, expanding the error distributions in the X and Y axes to ± 8 mm, accompanied by notable fluctuations in the Z‐axis under loading conditions. For the large circle trajectory (Circle #2), the RBRBL configuration showed even greater structural instability; under the 500 g load, the coordinate alignment degraded significantly in the X and Y axes, and the system struggled to maintain height stability, leading to an extreme peak downward deflection approaching −15 mm in the Z‐axis. Although the inclusion of two mvsRJs theoretically enhances the capability to execute circular trajectories, the placement of an mvsRJ behind an mvsBJ introduced additional Z‐axis fluctuations, reducing overall structural rigidity.

The BRBBL configuration, in contrast, demonstrated consistent and exceptional stability during both straight‐line and circular trajectories. Crucially, as evidenced in Figure 13H, the Z‐axis error box plots for both unloaded and loaded conditions remained remarkably compressed, with the total vertical deviation tightly restricted within ± 2 mm across all paths. This minimal vertical variance highlights its suitability for applications requiring precise height preservation. However, two notable pose changes were observed at specific points in each circular trajectory, resulting in discrete trajectory gaps larger than those seen in the RBRBL configuration.

Alternatively, the RRRBL configuration excelled in achieving complete circular trajectories (Figure 13K). Across both small and large loops, the tracking error medians for the X, Y, and Z axes were perfectly locked near 0 mm, with the main interquartile ranges bounded within a high‐precision band of ± 4 mm, showing no kinematic gaps and low deflection under the 500 g load. However, this configuration showed poor performance in executing straight‐line trajectories, making it less versatile compared to the other configurations. When comparing the loaded and unloaded conditions across all three configurations, no catastrophic growth in trajectory errors was observed, indicating that the pressure‐dependent compensation framework is reliable in managing steady‐state variations in external loading.

To rigorously evaluate path repeatability and controller robustness against unmodeled soft deflections, each trajectory tracking configuration was subjected to five independent experimental trials (*n* = 5). As systematically illustrated in the overhauled tracking results (Figure 13C–J), the continuous curves depict the calculated statistical Mean path across all five successful repetitions, seamlessly enveloped by a continuous shaded error band representing the Standard Deviation (± SD). The precise quantitative tracking errors (expressed as Mean ± SD) are directly annotated within each respective subplot. For the 4‐DoF RBRBL straight‐line path, the inter‐trial variability maintained strict boundaries, yielding errors of 1.78 ± 1.55 mm (X), 0.43 ± 0.69 mm (Y), and 1.51 ± 1.30 mm (Z) when unloaded, and 0.65 ± 0.72 mm (X), 0.23 ± 0.39 mm (Y), and 1.91 ± 1.01 mm (Z) under the 500 g load. For the 3‐DoF BRBBL configuration, the unloaded errors were 0.95 ± 0.62 mm (X), 0.27 ± 0.48 mm (Y), and 2.28 ± 2.09 mm (Z), while the 500 g loaded errors adjusted to 1.80 ± 0.89 mm (X), 0.21 ± 0.42 mm (Y), and 2.79 ± 1.62 mm (Z). For the complex multi‐DoF circular trajectories executed by the 4‐DoF RRRBL configuration, the unloaded errors were bounded at 2.15 ± 1.81 mm (X), 2.35 ± 2.10 mm (Y), and 2.31 ± 1.93 mm (Z); under the 500 g loaded circular state combining both small and large loops, the global absolute errors maintained a highly steady distribution of 2.33 ± 2.29 mm (X), 2.87 ± 2.75 mm (Y), and 1.84 ± 1.78 mm (Z). Concurrently, the adjacent box plots (Figure 13E,H,K) plot the comprehensive distribution of the absolute tracking errors relative to the geometric ground truth. These tightly constrained shaded variance profiles and bounded standard deviations mathematically demonstrate the exceptionally low inter‐trial variance and robust predictability of the proposed pressure‐dependent compensation control framework.

In summary, the experimental results confirm that the proposed control framework successfully facilitates motion planning and trajectory control across all tested configurations by accounting for factors such as self‐weight, external loads, and component deflections. Each configuration exhibits unique strengths and limitations within these tested environments: the RBRBL configuration is effective in minimising gaps caused by pose changes, the BRBBL configuration excels in maintaining height stability, and the RRRBL configuration achieves superior performance in circular trajectories. These findings underscore the baseline adaptability and robustness of the co‐bot system under specific operational conditions and task requirements. However, it is important to note that the current control architecture operates in an open‐loop fashion, meaning that critical non‐linear factors such as material hysteresis, long‐term silicone fatigue, and dynamic structural oscillations remain outside the compensation loop. Consequently, while the system demonstrates customizable rigidity and inherently safe interaction under steady‐state conditions, the claims of versatility are primarily bounded by these quasi‐static validation environments. Future iterations will prioritize closed‐loop feedback and sensory integration to mitigate these limitations and expand the system's applicability to highly dynamic tasks.

## Author Contributions

**Wenlong Gaozhang**: conceptualization, methodology, software, validation, formal analysis, writing – original draft, writing – review and editing, data curation, visualization, investigation. **Yue Li**: methodology, software, formal analysis, validation. **Jialei Shi**: methodology, software, formal analysis, validation. **Agostino Stilli**: conceptualization, methodology, supervision, visualization. **Yaxi Wang**: methodology, software, formal analysis, validation, investigation. **Kaspar Althoefer**: conceptualization, writing – review and editing. **Helge A. Wurdemann**: conceptualization, methodology, supervision, resources, visualization, writing – review and editing, funding acquisition.

## Funding

This work was supported by the Engineering and Physical Sciences Research Council (Grant Nos. EP/R037795/1, EP/S014039/1 and EP/V01062X/1), the European Commission's Horizon 2020 Research and Innovation Programme (project FourByThree under grant agreement No. 637095), the Royal Academy of Engineering (Grant No. IAPP18‐19∖264), and UCL Mechanical Engineering.

## Conflicts of Interest

The authors declare no conflicts of interest.

## Supporting information




**Supporting File 1**: advs77372‐sup‐0001‐SuppMat.docx.

**Supporting File 2**: advs77372‐sup‐0002‐MovieS1.mp4.

## Data Availability

Data sharing not applicable to this article as no datasets were generated or analysed during the current study.
