# Supplementary material for: A Modular Variable Stiffness Co‐Bot System Achieving Tasks Flexibility and Contact Compliance
Source: Adv Sci (Weinh). 2026 Aug 24;13(55):e77372. doi: 10.1002/advs.77372 (PMC13502353; doi:10.1002/advs.77372)
Supplement: Supplementary file 1 [file ADVS-13-e77372-s002.docx]

Supplementary Material for

**A modular variable stiffness co-bot system**

**achieving tasks flexibility and contact compliance**

Wenlong Gaozhang *et al.*

Corresponding author: Helge Wurdemann, h.wurdemann@ucl.ac.uk

**The file includes:**

Methods (Section S1, Section S2, Section S3)

Figs. S1 to S3

Tables S1 to S3

Legends for movies S1

**Other Supplementary Materials for this manuscript includes the following:**

Movies S1

**Supplementary Methods**

**Section S1: Trajectory generation and control framework**

In the basic form of motion planning, we directly map actuation pressures to joint angles using the actuation model $f_{\theta}^{B}(\Delta P_{B})$ and $f_{\theta}^{R}(\Delta P_{R})$. As illustrated in Figure S1 (A), the desired trajectory is discretized into coordinate series $\boldsymbol{X}_{n}(t)$, $\boldsymbol{Y}_{n}(t)$, $\boldsymbol{Z}_{n}(t)$. A kinematic model based on the D-H convention is constructed for each configuration, allowing joint angles $\boldsymbol{\theta}_{Bn}(t)$ and $\boldsymbol{\theta}_{Rn}(t)$ to be calculated via inverse kinematics using the MATLAB Robotics System Toolbox. The corresponding actuation pressures ${\Delta\boldsymbol{P}}_{Bn}(t)$ and ${\Delta\boldsymbol{P}}_{Rn}(t)$ are then obtained from the actuation model we built before.

To evaluate the trajectory planning method, various trajectories (e.g., circular and square paths) are applied to a configurations RBRBL, with results shown in Figure S1 (B). The system executes the planned motions. Experimental validation reveals discrepancies between planned and executed trajectories. These errors are attributed primarily to gravity-induced self-gravity $G_{self}$ and end load $G_{L}$. Besides, the deflection of the mvsL contributes to the errors else well despite we set a $P_{L}$ to increase its stiffness. To address this, a compensation scheme is introduced. A pose-dependent factor $c_{pose}$ is derived through pose analysis to adjust pressure compensation $\Delta P_{pose}$ for gravitational effects. Additionally, based on mvsL’s deflection model, a deflection-related compensation pressure $\Delta P_{\delta_{L}}$ is computed. Both terms are integrated into the original pressure profiles to yield optimised pressures ${\Delta\boldsymbol{P}}_{Bn}^{Fi}(t)$ and ${\Delta\boldsymbol{P}}_{Rn}^{Fi}(t)$. An optional stiffness control input $P_{S}$ can be added for global stiffness adjustment.

**Section S2:** **Workspace analysis for co-bots with potential configurations.**

Based on the prototypes of three mvsBJs (B), three mvsRJs (R), and two mvsLs (L), and considering the maximum channel capacity $N=10$, a total of 24 valid co-bot configurations can be constructed (excluding those with fewer than three bending/rotating joints). These are summarised in Table S1. An initial classification of these configurations, based on their workspace characteristics, leads to six categories: Single Point, Single Line, Flat Surface, Curved Surface, Regular Volume, and Irregular Volume. Configurations belonging to the first four categories possess relatively limited or simple workspaces and are not further analysed, with the exception of the RRRB configuration, which uniquely achieves circular motion without changing its orientation. The focus of detailed analysis is therefore placed on configurations yielding Regular Volume, Irregular Volume, and RRRB. A significant advantage of the proposed modular co-bots over conventional soft robots is the presence of fixed bending or rotation axes in their joints (mvsBJs and mvsRJs), allowing traditional kinematic modeling methods to be applied. Leveraging this feature, we employed the MATLAB Robotics System Toolbox to simulate the co-bot designs and generate their digital twins.

***Construction of Digital Twins:*** To build accurate digital models, Denavit-Hartenberg (D-H) parameters were determined for each configuration based on the known component lengths: mvsBJ: 100 mm**;** mvsRJ: 60 mm**;** mvsL: 175 mm. The calculated D-H coefficients are provided in Table S2. Redundant configurations, such as BRBR (identical to BRB) and RBBR (identical to RBB), are excluded. Nine configurations are ultimately selected for simulation: RRRB, RRBB, RBBB, BRB, RBB, RBRB, BRRB, BBRB, BRBB.

***Workspace Estimation via Monte Carlo Simulation:*** The Monte Carlo method is employed to estimate the reachable workspaces. The procedure includes: Range Setting: Joint limits are set to ± 55°for mvsBJs and ± 60°for mvsRJs; Random Sampling: 20,000 samples of joint angles are generated within the defined limits; Forward Kinematics: End-effector positions for each sample are calculated; Point Cloud Formation: All reachable positions are recorded as a point cloud; Envelope and Volume Calculation: A bounding envelope is constructed to evaluate workspace shape and volume.

Figure S2 illustrates the simulation results: Left - Digital twin models of each configuration, with red rods (mvsL) and cylinders (mvsBJ/mvsRJ); Right - Blue point clouds represent reachable areas; pink volumes show minimum bounding envelopes with calculated volumes.

Additionally, three rectangular and three circular trajectories are superimposed to assess trajectory feasibility.

***Application-Specific Insights:*** Different configurations suit different tasks - RRBL, RRRBL, RRBBL: Offer a near-spherical workspace, ideal for continuous motion without orientation change; BRLBL, BBRBL: Extended reach in one direction, suitable for long-distance linear tasks; RBBBL: Strong Z-axis coverage, useful for vertical movements (e.g., stacking, aerial adjustments); BRBBL: Broad vertical range, suitable for large 3D manipulation.

**Section S3: Variable stiffness evaluation of co-bots with different configurations.**

The ability to regulate stiffness is a key feature of the proposed modular soft robotic system. Based on the module-level characterizations, applying a dedicated stiffness-regulating pressure $P_{S}$ enables continuous and reversible modulation of stiffness. Therefore, it is necessary to examine how this capability translates to co-bot systems assembled from multiple modules and actuated into various poses.

To evaluate system-level stiffness, we define a stiffness matrix:

$$S_{co}=\left( \begin{matrix} \frac{F_{X}}{\Delta d_{X}^{F_{X}}} & \frac{F_{Y}}{\Delta d_{X}^{F_{Y}}} & \frac{F_{Z}}{\Delta d_{X}^{F_{Z}}} \\ \frac{F_{X}}{\Delta d_{Y}^{F_{X}}} & \frac{F_{Y}}{\Delta d_{Y}^{F_{Y}}} & \frac{F_{Z}}{\Delta d_{Y}^{F_{Z}}} \\ \frac{F_{X}}{\Delta d_{Z}^{F_{X}}} & \frac{F_{Y}}{\Delta d_{Z}^{F_{Y}}} & \frac{F_{Z}}{\Delta d_{Z}^{F_{Z}}} \end{matrix} \right)$$

where $F_{i}$ represents the applied Cartesian force along axis $i$, and $\Delta d_{j}^{F_{i}}$ denotes the resulting displacement along axis $j$ caused by force $F_{i}$. Each entry in the matrix reflects directional stiffness, allowing comprehensive characterisation of the co-bot’s mechanical response.

To measure this matrix experimentally, we applied known forces $F_{X}$, $F_{Y}$, and $F_{Z}$ to the co-bot’s end-effector using a traditional manipulator (Franka Emika Panda). A high-precision force/torque sensor (IIT F/T 17) captured the applied loads, while a 2-meter non-stretchable wire ensured consistent force direction. The corresponding 3D displacements were tracked using an NDI Aurora electromagnetic system.

To examine the pose dependency of stiffness, each co-bot configuration (RRRBL, RBRBL, BRBBL) was actuated into multiple typical poses using predefined pressure inputs, listed in Supplementary Table S3. In addition, to evaluate the effect of variable stiffness control, a regulating pressure $P_{S}=0.5\times{10}^{5}$ Pa was uniformly applied across all configurations and poses. The stiffness results are visualized in Supplementary Figure S3. Panel (A) shows the experimental setup. Panels (B–G) illustrate the stiffness matrix results using a spherical mapping method: starting from a unit sphere, each directional stiffness element deforms the sphere accordingly. The small, light-colored shapes represent the base stiffness without $P_{S}$, while the larger, darker shapes illustrate the enhanced stiffness under $P_{S}=0.5\times{10}^{5}$ Pa. The data reveal several key trends: The RRRBL configuration consistently exhibits the highest baseline stiffness, particularly in the Z-axis direction. For the same configuration, stiffness varies with pose. As the end-effector moves further from the base, overall stiffness tends to decrease, though Z-direction stiffness may increase. Stiffness regulation via $P_{S}$ reliably enhances performance, though non-uniformly. For example, in Pose 1 of the BRBBL configuration, Z-axis stiffness increases significantly, while X and Y enhancements are marginal. These results validate that the proposed co-bots can achieve directional, pose-dependent, and pressure-tunable stiffness, offering flexible mechanical adaptability for diverse soft robotic tasks.

**Supplementary Figures**

**Figure S1. Trajectory generation and control framework.** (A) Schematic of the trajectory generation and pressure-based control framework. The desired end-effector trajectory is discretized in Cartesian space and converted to joint space through inverse kinematics. Actuation pressures are derived from predefined pressure-angle mappings. (B) Experimental evaluation using various trajectories on the RBRBL configuration. Planned and actual trajectories are compared. Deviations are observed due to self-gravity, end-effector load, and mvsL deflection. Compensation terms $\Delta P_{pose}$ and $\Delta P_{\delta_{L}}$ are introduced to reduce trajectory errors. Final pressures ${\Delta\boldsymbol{P}}_{Bn}^{Fi}(t)$ and ${\Delta\boldsymbol{P}}_{Rn}^{Fi}(t)$ include these compensations and optional stiffness control $P_{S}$.

**Figure S2. Workspace simulation of nine selected co-bot configurations.** (A-I) Each subfigure presents the digital twin (left) and the corresponding reachable workspace point cloud with bounding envelope (right) for a specific configuration constructed from combinations of mvsBJs, mvsRJs, and mvsLs. Workspace estimation is performed via Monte Carlo simulation using 20,000 random joint configurations within joint angle limits of ±55° (bending) and ±60° (rotation). The calculated volumes of the minimum bounding envelopes are labeled in each case. Unreachable zones are annotated where observed. (J) The workspace analysis. RRBL, RRRBL, RRBBL: Offer a near-spherical workspace, ideal for continuous motion without orientation change. BRLBL, BBRBL: Extended reach in one direction, suitable for long-distance linear tasks. RBBBL: Strong Z-axis coverage, useful for vertical movements (e.g., stacking, aerial adjustments). BRBBL: Broad vertical range, suitable for large 3D manipulation. Configurations RRRBL, RBRBL, and BRBBL are selected for further evaluation due to their desirable workspace properties.

**Figure S3. Evaluation of stiffness performance across co-bot configurations and poses.** (A) Experimental setup for system-level stiffness characterisation. A Franka Emika Panda robot applies controlled forces to the end-effector of the modular soft co-bot using a non-stretchable wire, with a force/torque sensor (IIT F/T 17) and an NDI Aurora tracking system recording forces and displacements, respectively. (B–G) Directional stiffness results for different configurations and poses: RRRBL, RBRBL, and BRBBL. Each panel presents two stiffness profiles derived from the 3×3 stiffness matrix: one with no regulating pressure ($P_{S}=0$, light colour), and one with a regulating pressure of $P_{S}=0.5\times{10}^{5}$ Pa (dark colour). Spherical visualisation maps the directional stiffness values into an irregular 3D shape, illustrating stiffness anisotropy. Actuation pressures for each joint in the corresponding configuration and pose are labelled. Results confirm that stiffness varies with pose and configuration and can be modulated through the applied pressure $P_{S}$.

**Supplementary Tables**

| **Single point** | **Single line** | **Flat surface** | **Curve surface** | **Regular volume** | **Irregular volume** |
| --- | --- | --- | --- | --- | --- |
| RRR | BRR | BBR | RRB | RRBB | BRB |
| RRRR | BRRR | BBB | RBR | RBBB | RBB |
| ------ | ------ | BBRR | RRRB | ------ | RBRB |
| ------ | ------ | BBBR | RRBR | ------ | BRRB |
| ------ | ------ | BBBB | RBRR | ------ | BBRB |
| ------ | ------ | ------ | ------ | ------ | BRBB |
| ------ | ------ | ------ | ------ | ------ | BRBR |
| ------ | ------ | ------ | ------ | ------ | RBBR |

**Table S1. The categories of the workspace for all the potential co-bots configurations.**

| **Link** | **Link 1** | **Link 2** | **Link 3** | **Link 4** |
| --- | --- | --- | --- | --- |
| **Coefficient** | θ/d/a/α/o | θ/d/a/α/o | θ/d/a/α/o | θ/d/a/α/o |
| **RRRB** | 0/60/0/0/0 | 0/60/0/0/0 | 0/110/0/π/2/0 | 0/0/225/0/π/2 |
| **RRBB** | 0/60/0/0/0 | 0/110/0/π/2/0 | 0/0/100/0/π/2 | 0/0/225/0/0 |
| **RBBB** | 0/110/0/π/2/0 | 0/0/100/0/π/2 | 0/0/100/0/0 | 0/0/225/0/0 |
| **BRB** | 0/0/0/–π/2/0 | 0/335/0/π/2/0 | 0/0/225/0/π/2 | ------ |
| **RBB** | 0/110/0/π/2/0 | 0/0/270/0/π/2 | 0/0/225/0/0 | ------ |
| **RBRB** | 0/110/0/π/2/0 | 0/0/0/–π/2/0 | 0/160/0/π/2/0 | 0/0/225/0/π/2 |
| **BRRB** | 0/0/0/–π/2/0 | 0/110/0/0/0 | 0/110/0/–π/2/0 | 0/0/225/0/–π/2 |
| **BBRB** | 0/0/100/0/π/2 | 0/0/0/π/2/π/2 | 0/160/0/–π/2/0 | 0/0/225/0/π/2 |
| **BRBB** | 0/0/0/π/2/π | 0/160/0/π/2/0 | 0/0/100/0/π/2 | 0/0/225/0/0 |
| **BRBR** | 0/0/0/–π/2/0 | 0/160/0/π/2/0 | 0/0/0/π/2/π | 0/285/0/0/0 |
| **RBBR** | 0/110/0/π/2/0 | 0/0/100/0/π/2 | 0/0/0/π/2/π/2 | 0/285/0/0/0 |

**Table S2. The D-H matrix coefficient of the co-bot in the workspace analysis.**

|  | **RRRBL** | | | | | **RBRBL** | | | | | **BRBBL** | | | | |
| --- | --- | --- | --- | --- | --- | --- | --- | --- | --- | --- | --- | --- | --- | --- | --- |
| **Pose** | R | R | R | B | L | R | B | R | B | L | B | R | B | B | L |
| 1 | 1 | 1 | 1 | 1 | 0.5 | 1 | 1 | 1 | 1 | 0.5 | 1 | 1 | 1 | 1 | 0.5 |
| 2 | - | - | - | - | - | -1 | -1 | 1 | 1 | 0.5 | 1 | 1 | 1 | -1 | 0.5 |
| 3 | - | - | - | - | - | - | - | - | - | - | 1 | 1 | -1 | -1 | 0.5 |

**Table S3. Typical poses using predefined pressure inputs for stiffness evaluation.**

**Supplementary Movies**

**Movie S1.** Scene 1: An introduction to the complete prototype family, comprising two modular variable-stiffness Bending Joints (mvsBJs), modular variable-stiffness Rotating Joints (mvsRJs), and two modular variable-stiffness Links (mvsLs). The assembly process is demonstrated, showcasing how the co-bot modules can be rapidly and securely connected using the fast-connection design. The end-effector trajectory control is illustrated, along with real-time displacement tracking in the X, Y, and Z directions. Corresponding error curves for each axis are also presented to evaluate the control accuracy. Scene 2: Full experimental recording of Application Scenario 1 – Bedside Feeding Assistance for Patients, including both autonomous feeding and physical human interaction. The actuation pressure of each module is recorded throughout the process, alongside the contact force measured at the end-effector. These data help analyse the interaction dynamics and effectiveness of the proposed co-bot system in assistive healthcare tasks. Scene 3: Full experimental recording of Application Scenario 2 – Industrial Chamfering Assistance, featuring a comparison between the proposed co-bot and a conventional bench vice during the chamfering process. Displacement and contact force data are recorded to estimate the stiffness of the co-bot holding platform. A follow-up inspection of the chamfered edges is performed to evaluate the quality, efficiency, and feasibility of using the co-bot in industrial support tasks.
